# Targeted ASO-mediated *Atp1a2* knockdown in astrocytes reduces SOD1 aggregation and accelerates disease onset in mutant SOD1 mice

**DOI:** 10.1371/journal.pone.0294731

**Published:** 2023-11-28

**Authors:** Abhirami K. Iyer, Kathleen M. Schoch, Anthony Verbeck, Grant Galasso, Hao Chen, Sarah Smith, Anna Oldenborg, Timothy M. Miller, Celeste M. Karch, Azad Bonni

**Affiliations:** 1 Department of Neuroscience, Washington University School of Medicine, St. Louis, Missouri, United States of America; 2 Department of Psychiatry, Washington University School of Medicine, St. Louis, Missouri, United States of America; 3 Department of Neurology, Washington University School of Medicine, St. Louis, Missouri, United States of America; 4 Hope Center for Neurological Disorders, Washington University School of Medicine, St. Louis, Missouri, United States of America; 5 Neuroscience and Rare Diseases, Roche Pharma Research and Early Development (pRED), Roche Innovation Centre Basel, Basel, Switzerland; Medical College of Georgia at Augusta University, UNITED STATES

## Abstract

Astrocyte-specific ion pump α2-Na^+^/K^+^-ATPase plays a critical role in the pathogenesis of amyotrophic lateral sclerosis (ALS). Here, we test the effect of *Atp1a2* mRNA-specific antisense oligonucleotides (ASOs) to induce α2-Na^+^/K^+^-ATPase knockdown in the widely used ALS animal model, SOD1*G93A mice. Two ASOs led to efficient *Atp1a2* knockdown and significantly reduced SOD1 aggregation *in vivo*. Although *Atp1a2* ASO-treated mice displayed no off-target or systemic toxicity, the ASO-treated mice exhibited an accelerated disease onset and shorter lifespan than control mice. Transcriptomics studies reveal downregulation of genes involved in oxidative response, metabolic pathways, trans-synaptic signaling, and upregulation of genes involved in glutamate receptor signaling and complement activation, suggesting a potential role for these molecular pathways in de-coupling SOD1 aggregation from survival in *Atp1a2* ASO-treated mice. Together, these results reveal a role for α2-Na^+^/K^+^-ATPase in SOD1 aggregation and highlight the critical effect of temporal modulation of genetically validated therapeutic targets in neurodegenerative diseases.

## Introduction

Amyotrophic lateral sclerosis (ALS) is a progressive neurodegenerative disease ultimately resulting in death within 3–5 years of disease onset. The majority of ALS cases are sporadic; however, 5–10% of ALS cases are caused by rare mutations [1]. Mutations in the gene encoding *superoxide dismutase 1* (*SOD1*) comprise the first reported genetic cause of ALS [2]. Over 180 *SOD1* mutations have been reported to date, and they account for 15–20% of familial and ∼3% of sporadic ALS cases [1]. Mice expressing human familial ALS-linked *SOD1* mutations develop age-dependent motor neuron degeneration and show the presence of cytoplasmic inclusions and protein aggregation as observed in ALS patients [3]. These studies have suggested that misfolding and increased aggregation of mutant SOD1 represents a toxic gain of function that contributes to disease pathogenesis and decreased patient survival time [4–7]. Furthermore, SOD1*G93A mice also exhibit non-cell autonomous disease mechanisms, implicating glia as major drivers of disease progression and/or initiation [8].

Mutant SOD1*G93A astrocytes contribute to motor neuron degeneration via a non-cell autonomous mechanism. One such possible mechanism involves upregulation of a protein complex of ion-pump α2-Na^+^/K^+^-ATPase and membrane cytoskeletal protein α-adducin [9]. α2-Na^+^/K^+^-ATPase is predominantly expressed in astrocytes in the adult brain and shows perisynaptic localization and co-distribution with glutamate transporters [10–12]. The levels of α2-Na^+^/K^+^-ATPase and α-adducin proteins are also increased in the spinal cord of sporadic and familial ALS patients [9]. RNAi knockdown of *Atp1a2 or Add1* mRNA in primary SOD1*G93A astrocytes protects co-cultured motor neurons from cell death and preserves dendrite morphology [9]. Furthermore, SOD1*G93A mice with heterozygous knockout of *Atp1a2* exhibit significantly delayed disease onset, slowed disease progression, and extended survival [9]. Together, these data suggest that increased activation of α-adducin/α2-Na^+^/K^+^-ATPase complex represents a glial cell-intrinsic mechanism of non-cell autonomous neurodegeneration in ALS and support *Atp1a2* as a potential therapeutic target in ALS.

Disease-modifying therapies are currently limited for ALS. Riluzole, an anti-glutaminergic drug, and edaravone, an antioxidant, confer only a modest extension of life [13]. However, there is an emerging line of therapeutics to treat ALS via the selective manipulation of aberrant genes using antisense oligonucleotides (ASOs), including the recent FDA-approved Tofersen, which lowers SOD1 mRNA and slows clinical decline with earlier treatment initiation [14–16]. Nusinersen, another FDA-approved ASO designed to promote full-length SMN2 protein production and complement SMN1 loss in the pediatric motor neuron disease, spinal muscular atrophy [17, 18], and the promising preclinical phase 1–2 and 3 trial data for Tofersen in ALS [15, 16, 19] have ushered in the development of additional ASO-based therapies for neurological diseases [20–22].

In this study, we have investigated the suitability of inhibiting astrocytic α2-Na^+^/K^+^-ATPase by ASOs as a potential therapeutic approach in ALS. Intracerebroventricular injection of SOD1*G93A mice prior to disease onset with *Atp1a2*-specific ASOs led to efficient *Atp1a2* reduction and significantly reduced SOD1 aggregation in the spinal cord. However, *Atp1a2* knockdown in SOD1*G93A mice led to accelerated disease onset and shorter lifespan compared to animals receiving control ASOs. Transcriptomics studies indicate *Atp1a2-*knockdown induced gene alterations in trans-synaptic and glutamate receptor signaling, oxidative response, complement activation, and metabolic pathways as potential mechanisms for observed outcomes. Collectively, these results suggest that α2-Na^+^/K^+^-ATPase may be involved in SOD1 aggregation and a number of diverse molecular pathways contributing to disease pathology in ALS. However, further studies are warranted to determine the optimal time window for ASO-based intervention to therapeutically target α2-Na^+^/K^+^-ATPase in ALS.

## Materials and methods

### Animals

All animal experiments in the study were performed per institutional guidelines and have been approved by the Institutional Animal Care and Use Committee of Washington University School of Medicine, St. Louis. Male B6SJL-Tg (SOD1*G93A)1Gur/J mice (Jackson Laboratory; stock no.: 002726) were mated with female B6SJLF1/J mice (Jackson Laboratory; stock no.: 100012) to establish the SOD1*G93A mouse colony. All mice used in this study were from F1 litters and were verified for human *SOD1* transgene expression using genotyping PCR. Both male and female mice from independent litters were used in this study and animals were age- and sex-matched for every experiment.

### *Atp1a2*-specific locked nucleic acid modified antisense oligonucleotides (LNA ASOs) design and production

Antisense LNA GapmeRs targeting different regions of the 3’-UTR of mouse *Atp1a2* (NM_178405.3) were designed using the Qiagen GeneGlobe Antisense LNA GapmeR designer purchased from Qiagen, sequences of ASOs used for *in vitro* and *in vivo* experiments are provided in S12 Table. ASOs were designed as short 15-16mer, single-stranded oligonucleotides comprised of a central gap of LNA-free DNA flanked on either sides by LNA-modified residues [20]. ASOs used for *in vitro* experiments were purified by standard desalting. ASOs used for *in vivo* experiments were purified by anion-exchange high performance liquid chromatography (HPLC), desalted, and lyophilized as sodium salt by the manufacturer. The identity of each ASO was confirmed by electrospray ionization mass spectrometry (ESI-MS) with a purity of >85%.

### Screening of *Atp1a2*-specific ASOs in primary astrocytes

Primary murine astrocytes used in this study were generated from the cortices of post-natal day 2 (P2) pups (n = 10–12) from either CD1 wild-type strain (code: 022, #24101187 from Charles River Laboratories) or SOD1*G93A mice as previously described [23, 24]. Primary murine astrocytes were nucleofected with a non-targeting negative control A (ctrl ASO) or *Atp1a2*-specific ASOs at several concentrations using the Amaxa™ Basic Nucleofector™ Kit for Primary Mammalian Glial Cells (Lonza, VPI-1006) or P3 Primary Cell 4D-Nucleofector^TM^ X Kit (Lonza, V4XP-3032) following the manufacturer’s protocol. Briefly, primary mouse astrocytes were harvested on day 7 in culture by trypsinization and, after determining cell counts, cells were centrifuged at 100xg for 10 minutes at room temperature. 1-2x10^6^ cells were used for each nucleofection reaction in 100μl room temperature Nucleofector™ solution (or P3 Primary Cell Nucleofector^TM^ Solution and supplement (18μl/100μl of Nucleofector™ solution). The cells were immediately transferred to cuvettes and nucleofection was performed using the recommended T-020 optimal Nucleofector™ program in the Amaxa Nucleofector™ II device (or CL-133 program optimized for primary mouse astrocytes in the 4D Nucleofector™ unit) ensuring minimal time spent by cells in Nucleofector™ solution. Following nucleofection, 500μl of pre-warmed culture media was added to each cuvette and the cells were transferred to 12- or 24-well plates precoated with PDL. For each ctrl ASO and *Atp1a2*-specific ASO tested, three independent nucleofection experiments were performed with replicates as indicated in each experiment. For IC_50_ measurement, primary mouse astrocytes were nucleofected with a dose range of 10-1500nM for ASOs 1 and 3 and then seeded into 24-well plates precoated with PDL. Astrocytes were monitored for their viability post-nucleofection and were harvested either 48, 72, or 96 hours (h) later for target knockdown mRNA, protein analyses, and RNA-sequencing, respectively.

### Cell viability assay

Primary mouse astrocytes were nucleofected with control or *Atp1a2*-specific ASOs and then seeded into PDL-coated 96-well microplates for fluorescence-based assays (ThermoFisher Scientific, M33089) and cultured for 48h. After 48h, 1/10^th^ volume of alamarBlue™ (ThermoFisher Scientific, DAL1025) cell viability reagent was added to each well and the plate was incubated for 4 hours, after which, fluorescence was measured at 600nm in a Biotek Synergy HTX Multi-mode Microplate Reader. The assay was performed in 8 replicates per treatment at each of the concentrations tested.

### Intracerebroventricular (ICV) injections for in vivo studies

Control or *Atp1a2*-specific ASOs were delivered centrally via ICV single bolus injection as previously described [25]. Briefly, animals were anesthetized using 4% isoflurane. The mouse head was shaved, then placed within a stereotax frame under constant 2% inhalant isoflurane and temperature regulation via heating pad throughout the procedure. The surgical area was cleaned followed by an incision on the skin from the base of the neck to the area in between the eyes. Hamilton syringe needles (Hamilton, 10μL Gastight Syringe Model 1701 RN, 7653-01- and 22s-gauge, Small Hub RN Needle, 7758–03) preloaded with desired concentration of ctrl ASO or *Atp1a2* LNA ASO in a total volume of 10μl were aligned 1.0mm laterally to the right and 0.3mm anterior from bregma. The needle was inserted into the skull until the bevel was flush with top of the skull and then lowered -3.0mm. After a 2–3-minutes (mins) delay, ctrl ASO or *Atp1a2*-specific ASO was administered at a rate of 1μl/s into the lateral ventricle. The needle remained in place for an additional 2-3mins after delivery and then withdrawn at a rate of 1mm/s. The incision was sutured, and antibiotic ointment was applied. The mouse was allowed to recover on a heated pad until ambulatory with most mice recovering 20mins to 2h after ICV injection. The mice were examined everyday thereafter for pain, discomfort, and infections. All ICV injections were performed in a blinded manner such that the person processing the tissues and carrying out downstream analyses was blinded to the agent administered in each mouse until all the experimental analyses were completed.

### *In vivo* dose response and duration of action of Atp1a2-specific ASO after single bolus ICV administration

For the preliminary dose response study, ICV injections were performed in female SOD1*G93A mice at 6–8 weeks of age and prior to disease onset received 25, 50, or 75μg of either control or *Atp1a2* ASO. Animals were sacrificed 2 weeks post-ICV injections using ketamine/xylazine cocktail. Mice were perfused with 0.03% heparin (Sigma, H3149) in PBS. After perfusion, blood collection via cardiac puncture was performed to isolate serum by centrifugation at 1500xg for 10mins at 4˚C and sera in the supernatant was stored at -80˚C. Central nervous system (CNS) tissue was dissected into cortical/midbrain (CX/MB), cerebellum/brainstem (CB/BS), and spinal cord (SC) regions and were harvested, flash frozen in liquid nitrogen, and stored at -80˚C until further analyses. Liver, spleen, and skeletal muscle tissue were also harvested, flash frozen, and stored as mentioned. To determine the duration of action of the single bolus administered ASOs, female SOD1*G93A mice at 6–9 weeks of age received either ctrl ASO (25 and 50μg), ASO1 (50μg), or ASO3 (25μg) via ICV injection. Mice were sacrificed at 2, 4, 8, or 12 weeks after ASO administration, and tissues were harvested and stored as described above.

### Survival outcomes and motor function assessment after *Atp1a2* ASO administration

ICV injections were performed to deliver ctrl ASO (25 and 50μg), ASO1 (50μg), or ASO3 (25μg) to SOD1*G93A mice of both sexes from independent litters at 5–8 weeks of age. All groups of SOD1*G93A mice were weighed 3 times per week until symptom onset, at which time mice were monitored and weighed every day at a similar time of the day to determine humane endpoint. For motor function assessment, mice were assigned neurological scores (NS) following protocol developed by ALS Therapy Development Institute for SOD1*G93A mice to perform an unbiased assessment of onset of paresis, progression, and severity of paralysis [26]. Briefly, on days of weighing, mice were suspended by their tails, monitored for gait, and placed on their sides after onset of paresis and NS were assigned for each mouse on a scale from 0 to 4, such that NS0 indicates presymptompatic, NS1 for first symptoms such as abnormal splay or trembling during tail suspension, NS2 for onset of paresis, NS3 for paralysis and NS4 for humane endpoint. Disease onset in SOD1*G93A mice was marked as the postnatal day when animals had lost 10% of their peak body weight. Animals were sacrificed at end-stage: defined for SOD1*G93A mice as the inability to right themselves within 30s when placed on their side [27]. CNS, peripheral tissues, and mouse sera were collected and stored as described above.

### RNA extraction and quantitative PCR

Total RNA was extracted from harvested mouse tissues using RNeasy Mini Kit (Qiagen, 74104) and cDNA was prepared from ∼0.5–1μg RNA using SuperScript™ III Reverse Transcriptase (ThermoFisher Scientific, 18080–093) per manufacturer’s protocols. qPCR was performed in triplicates with iTaq™ Universal SYBR® Green Supermix (Bio-Rad, 1725121) on the QuantStudio 6 Flex Real-Time PCR System (Applied Biosystems). Fold change in gene expression was calculated relative to housekeeping gene *Gapdh* using the 2^–ΔΔCt^ method. Sequences for primers used in this study are reported in S1 Table.

### Measurement of SOD1 and p62 aggregation in detergent-insoluble fraction from spinal cord

Detergent insoluble SOD1 aggregates were measured in spinal cord homogenates as previously described [5, 28]. Briefly, spinal cords from control or *Atp1a2* ASO-treated SOD1*G93A mice were weighed and mixed with 10 volumes of 1x TEN buffer (10mM Tris, pH-7.5; 1mM EDTA, pH-8.0; 100mM NaCl; 1x protease inhibitor) and homogenized with a probe sonicator (Fisherbrand™ Model 120 Sonic Dismembrator). The homogenate was centrifuged at 800xg for 10m at 4˚C. The crude supernatant was transferred to new tubes, mixed with 200μl 1x extraction buffer 2 (10mM Tris, 1mM EDTA, 100mM NaCl, 0.5% Nonidet P40, and 1x protease inhibitor mixture) and sonicated to resuspend; the pellet from this step was discarded. The extract was then centrifuged at 100,000xg for 30m at 4˚C in the Beckman Optima™ MAX-XP tabletop ultracentrifuge (MLA 130 fixed angle rotor) to separate insoluble pellet (P2) from soluble supernatant (S1). S1 supernatant was saved for analysis and the P2 pellet was resuspended in buffer 3 (10mM Tris, 1mM EDTA, 100mM NaCl, 0.5% Nonidet P40, 0.25% SDS, 0.5% deoxycholic acid, and 1× protease inhibitor mixture) by sonication. After estimating protein concentrations using BCA assay, S1 (5μg) and P2 (20μg) fractions were boiled for 5m at 90˚C and resolved in Criterion TGX precast gels (Bio-Rad, 5671123) at 125V, followed by transfer to 0.45μm pore size Immobilon-P PVDF Membrane (Millipore, IPVH00010) overnight at 50mA. After transfer, membranes were rinsed with Milli-Q® water, stained with PonceauS staining solution for 10mins, and rinsed with Milli-Q® water to remove background stain and imaged for total protein normalization. The blots were then probed with rabbit anti-SOD1 antibody (1:1000; Sigma, SAB5200083) and mouse anti-SQSTM1/p62 (1:500, monoclonal antibody, clone 2C11, Thermo Scientific, H00008878-M01) followed by probing with HRP-conjugated anti-rabbit or anti-mouse secondary antibodies. The blots were visualized using SuperSignal™ West Pico PLUS Chemiluminescent Substrate (Thermo Fisher, 34577). Intensities of SOD1, p62 bands from S1 and P2 fractions and PonceauS from S1 were quantified using ImageJ, and the graphs showing their relative expression were quantified using GraphPad Prism (version 9.5.0).

### Immunoblotting for α2-Na^+^/K^+^-ATPase knockdown verification

Primary mouse astrocytes treated with control or *Atp1a2* ASO or spinal cord from control or *Atp1a2* ASO-treated mice were lysed in 50mM Tris-HCl (pH-7.4), 100mM NaCl, 1mM EDTA, 1mM DTT, and protease inhibitor cocktail. Protein estimation was performed by Pierce™ BCA Protein Assay Kit (ThermoFisher Scientific, 23227) following manufacturer’s protocol. Lysates were then sonicated for 6 pulses of 30s each with 30s intervals on an ice bath in cold room to enable solubilization of membrane protein, α2-Na^+^/K^+^-ATPase. Lysates were boiled for 10mins at 37˚C and 5μg lysates were then loaded on 7% resolving SDS-PAGE gel at 100V, transferred to 0.2μm pore size nitrocellulose membrane (Amersham™ Protran® Western blotting membranes, GE10600011) for 2h at 200mA, and probed with rabbit anti-sodium pump subunit alpha-2 antibody (used at 1:20000; Millipore Sigma, AB9094-I) and anti-GAPDH antibody (used at 1:1000, Santa Cruz Biotechnology, sc-32233) followed by HRP-conjugated anti-rabbit secondary antibody (Peroxidase AffiniPure Goat Anti-Mouse IgG (H+L), Jackson Immunoresearch Laboratories, 115-035-003). The blots were developed using ECL-based chemiluminescence method. Intensities of α2-Na^+^/K^+^-ATPase and GAPDH bands were measured using ImageJ.

### Transcriptomic analysis

RNA-sequencing was performed on total RNA extracted from SOD1*G93A primary mouse astrocytes nucleofected with either ctrl ASO or *Atp1a2* ASO for 96h and in end-stage spinal cord samples from control, *Atp1a2* ASOs 1 and 3 treated SOD1*G93A mice. Samples were prepared according to library kit manufacturer’s protocol, indexed, pooled, and sequenced on an Illumina NovoSeq. Basecalls and demultiplexing were performed with Illumina’s bcl2fastq software and a custom python demultiplexing program with a maximum of one mismatch in the indexing read. RNA-seq reads were then aligned to the Ensembl release 76 primary assembly with STAR version 2.5.1a [29]. Gene counts were derived from the number of uniquely aligned unambiguous reads by Subread:featureCount version 1.4.6-p5 [30]. Sequencing performance was assessed for the total number of aligned reads, total number of uniquely aligned reads, and features detected. The ribosomal fraction, known junction saturation, and read distribution over known gene models were quantified with RSeQC version 2.6.2 [31].

All gene counts were then imported into the R/Bioconductor package EdgeR [32] and TMM normalization size factors were calculated to adjust samples for differences in library size. Ribosomal genes and genes not expressed in the smallest group size minus one sample greater than one count-per-million were excluded from further analysis. The TMM size factors and the matrix of counts were then imported into the R/Bioconductor package Limma [33]. The performance of all genes was assessed with plots of the residual standard deviation of every gene to their average log-count with a robustly fitted trend line of the residuals. Differential expression analysis was then performed to analyze for differences between conditions. Gene Ontology, KEGG, and WikiPathways (species: Mus musculus, year queried: 2019) were used to derive functional annotations of the differentially expressed genes into pathways.

### Immunohistochemistry of spinal cord tissue

Flash frozen spinal cord tissues from end-stage control, *Atp1a2* ASO1 or ASO3 treated mice were fixed with 4% PFA for 16h followed by transfer to 30% sucrose in PBS for 24-48h and embedding tissue on OCT. OCT embedded tissues were then sectioned in a chilled microtome at 40μm thickness, and the sections were stored in cryoprotectant solution in 24-well plates at -20°C until ready to be processed for immunohistochemical (IHC) staining. When ready to perform IHC, sections were washed three times with Tris-buffered saline (TBS) with 0.1% Triton X-100 (TBST) for 5m each wash, followed by blocking in 5% normal donkey serum (NDS) for 1h at room temperature. Following blocking, sections were incubated with the following primary antibodies in 5% NDS and TBST buffer–rabbit Alexa Fluor 594 Anti-NeuN (1:250, Abcam ab207279), rat anti-GFAP (monoclonal, 2.2B10, 1:200, Sigma-Aldrich 345860), goat anti-Choline Acetyltransferase (1:100, EMD Millipore, AB144P), and anti-misfolded SOD1 (mfSOD1, monoclonal, C4F6, 1:100, Medimabs, MM0070-2-P) at 4°C overnight. For ChAT and mfSOD1 IHC, antigen retrieval was performed on tissues prior to proceeding to staining protocol for 5m at 80°C, followed by blocking and staining in 5% NDS and 0.4% Triton X-100 containing TBST buffer. Following the primary antibody incubation, sections were washed three times with TBST for 5m each wash followed by blocking in 5% NDS TBST buffer for 30mins at room temperature. The sections were then incubated with fluorophore (Alexa Fluor 488 or 647) conjugated highly cross-adsorbed secondary antibodies raised in donkey (1:500). Images were acquired at 20x magnification in Keyence BZX all-in-one fluorescence microscope and scale bar indicates 100μm.

### Serum creatinine, aspartate aminotransferase and alanine aminotransferase measurements

Creatinine, aspartate, and alanine aminotransferases were measured in the serum of control and *Atp1a2-*specific ASO-treated SOD1*G93A mice by the Division of Comparative Medicine Research Animal Diagnostic Laboratory using the AMS Liasys 330 Clinical Chemistry System.

### Statistical analysis

All statistical analyses in this study were conducted using GraphPad Prism software. Details on sample sizes (n), statistical tests employed, and results of exact p-values are indicated in figure legends. Bar graphs are presented as the mean ± SD. Data distribution was assumed to be normal, but this was not formally tested.

## Results

### Design of locked nucleic acid (LNA) ASO gapmers targeting *Atp1a2*

**Fig 1:**
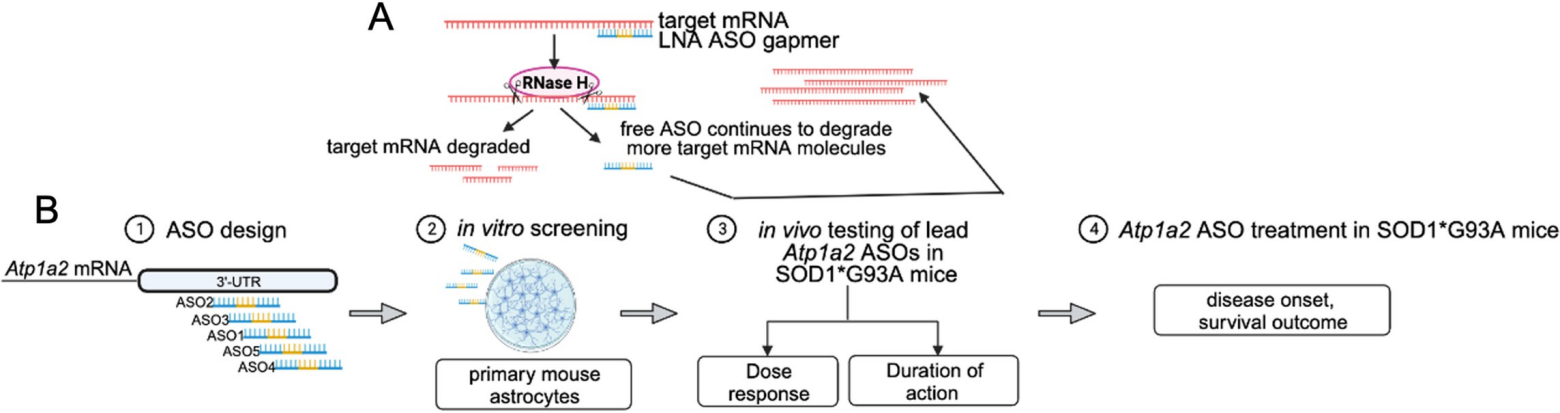
Study overview. (A) Mechanism of gene knockdown by locked nucleic acid (LNA) antisense oligonucleotides (ASO) employed in this study. (B) Workflow: (1) Five distinct LNA ASO GapmeRs targeting different regions of the 3’-UTR of mouse *Atp1a2* were designed, a non-targeting negative control LNA GapmeR (ctrl) was also used in this study to confirm *Atp1a2* knockdown-specific effects and rule out general response to LNA oligonucleotides; (2) the five ASOs were screened for their efficiency of *Atp1a2* knockdown using primary mouse astrocytes; (3) two ASOs and ctrl were then delivered in a single dose into the central nervous system of SOD1*G93A mice via intracerebroventricular (ICV) administration prior to disease onset to identify the optimal ASO concentration for target knockdown with minimal toxicity; (4) treatment of SOD1*G93A mice prior to disease onset with optimal ASO concentration to evaluate the impact of *Atp1a2* knockdown on disease onset and survival.

To investigate the therapeutic potential of targeting α2-Na^+^/K^+^-ATPase, we employed an ASO-based approach to induce knockdown of *Atp1a2* mRNA encoding α2-Na^+^/K^+^-ATPase (Fig 1A). The different isoforms of the catalytic α subunit of Na^+^/K^+^-ATPases are encoded by distinct genes [34, 35]. Using NCBI nucleotide BLAST, we performed pairwise sequence alignments and found that *Atp1a2* shares 77%, 81%, and 79% sequence identity with mRNAs encoding the α1 (*Atp1a1*), α3 (*Atp1a3*), and α4 (*Atp1a4*) subunits of Na^+^/K^+^-ATPase. The region from 4000 to 6227 in *Atp1a2* is unique containing the 3’-untranslated region (UTR) of *Atp1a2*. Since the 3’-UTR is a “hot-spot” for RNase-H dependent oligonucleotides [36, 37], the sequence from 4000 to 6227 bases of *Atp1a2* was used as input to design *Atp1a2*-LNA ASO gapmers. The top five ranked *Atp1a2-*specific LNA ASOs from this search (S2 Table) were evaluated *in vitro* for their ability to induce knockdown of *Atp1a2*. A negative control LNA oligonucleotide with the identical chemical modification and backbone as *Atp1a2-*specific LNA ASOs, but no known targets in the human, mouse, or rat genome was also included (ctrl ASO). Following *in vitro* assessment, two lead ASO compounds were advanced to *in vivo* testing to determine optimal dosage and length of sustained target knockdown after a single intracerebroventricular (ICV) administration. These experiments were then used to guide the ASO treatment study in SOD1*G93A ALS mice to determine the effect of *Atp1a2* knockdown on disease onset and survival outcome (Fig 1B).

### Identification of functional *Atp1a2* ASOs in primary murine astrocytes

**Fig 2:**
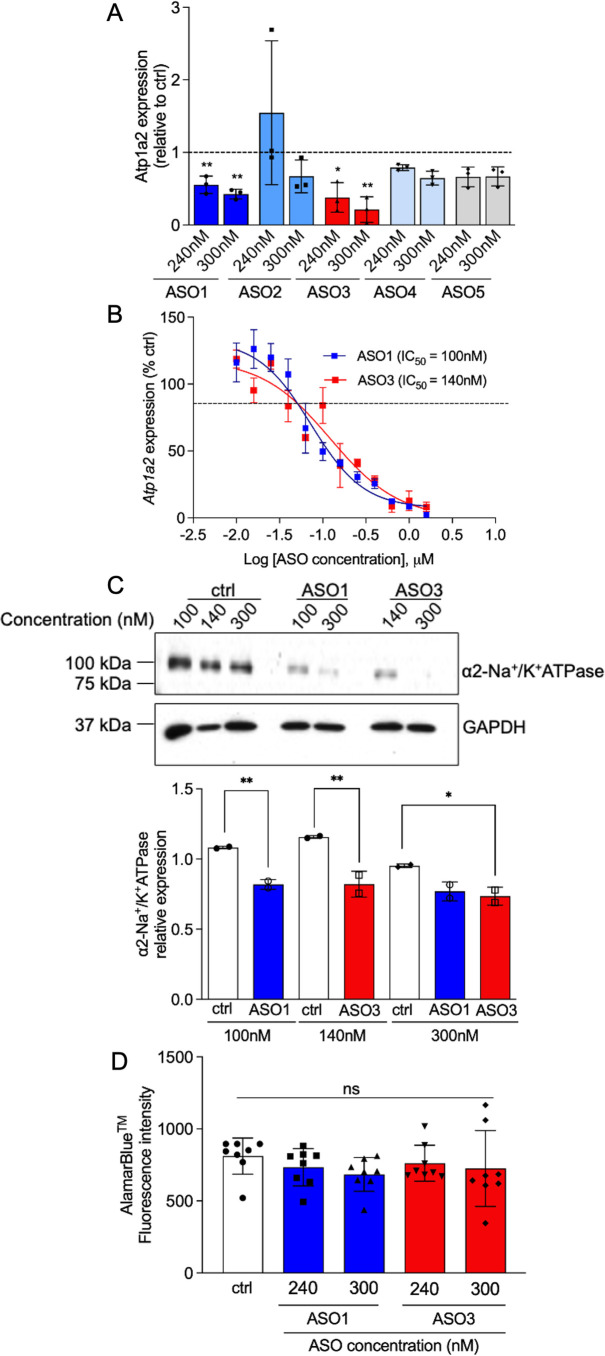
Identification of ASOs targeting *Atp1a2* that cause knockdown at the mRNA and protein levels with minimal cytotoxicity. Primary murine wild-type (WT) astrocytes were nucleofected with ctrl or *Atp1a2* ASOs at indicated concentrations for 48h (A, B and D) or 72h (C) and were then subjected to downstream analyses. Relative levels of *Atp1a2* mRNA in astrocytes nucleofected with *Atp1a2* ASO1, ASO3 or ctrl. Data shown here are fold change values from 3 independent experiments. One-way ANOVA with Dunnett’s multiple comparisons test. **p<0.01, *p<0.05. (B) Dose response curve for *Atp1a2* ASO1 and ASO3 in primary WT astrocytes, *Atp1a2* mRNA after ASO treatment was compared to ctrl-treated cells. (C) Immunoblot analyses of α2-Na^+^/K^+^-ATPase protein knockdown in *Atp1a2* ASO1 or ASO3-treated WT astrocytes. Representative image from 2 independent experiments. Quantitation of α2-Na^+^/K^+^-ATPase band intensities relative to that of GAPDH in *Atp1a2* ASO1, ASO3 or scrambled control nucleofected cells from 2 independent experiments. One-way ANOVA with Sidak’s multiple comparisons test, **p<0.01, *p<0.05 (D) Primary WT astrocytes were treated with alamarBlue™ cell viability reagent for 4h after 48h pretreatment with either control, *Atp1a2* ASO1 or ASO3. Fluorescence intensity is chemically reduced by metabolically active cells. N = 8 replicate wells for each condition. ns, not significant.

To identify ASOs that induce efficient knockdown of the target, control or *Atp1a2* ASOs were transfected into primary non-transgenic mouse astrocytes using nucleofection. Two ASOs, ASO1 and ASO3, resulted in ∼50–75% knockdown of *Atp1a2* in mouse astrocytes (Fig 2A). Both ASOs produced a statistically significant dose-dependent reduction in *Atp1a2* mRNA (Fig 2B). Neither ASO1 nor ASO3 induced knockdown of *Atp1a1*, suggesting that the two ASOs specifically induce knockdown of *Atp1a2* (S1A Fig). The levels of α2-Na^+^/K^+^-ATPase protein were significantly reduced in *Atp1a2* ASO-treated astrocytes compared with ctrl ASO-treated astrocytes (Fig 2C). To assess the impact of ASOs on cell toxicity, an alamarBlue viability assay was used to detect metabolically active cells. *Atp1a2* ASO-treated astrocytes had similar fluorescence measurements compared to the ctrl ASO-treated astrocytes (Fig 2D), illustrating the absence of overt toxicity of *Atp1a2* ASOs at the concentrations used in these experiments. Therefore, we prioritized the two candidate *Atp1a2*-specific ASOs, ASO1 and ASO3, which efficiently target *Atp1a2* mRNA with minimal cytotoxicity.

### Dose response and duration of action of *Atp1a2* ASOs in SOD1*G93A mice

**Fig 3:**
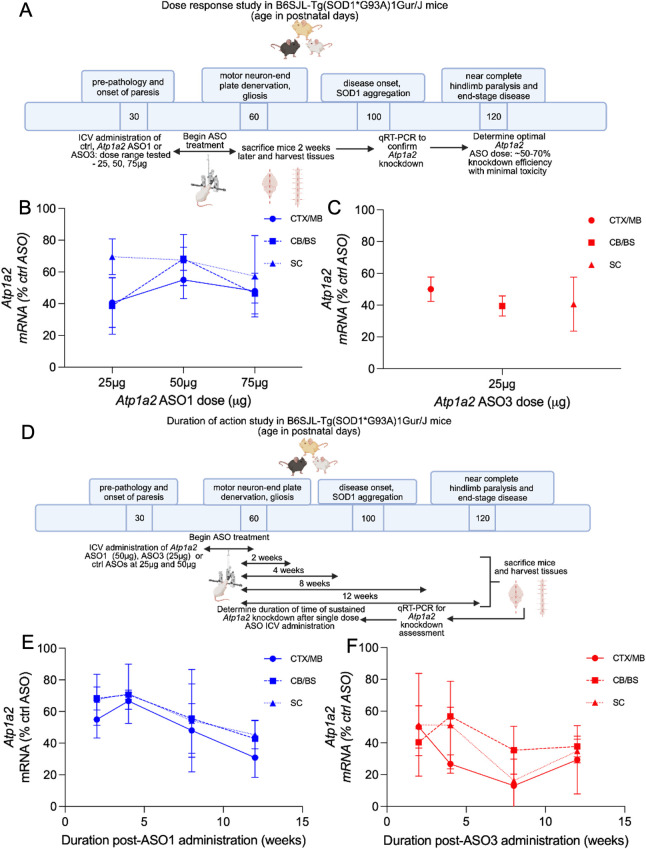
*In vivo* validation of *Atp1a2* knockdown. (A) Overview of dose response study (n = 3–4 female mice/group, 6–9 weeks old mice, prior to disease onset). B-C. *Atp1a2* mRNA levels in CNS regions 2 weeks after ICV treatment with ASO1 (B) or ASO3 (C), represented as percentages of that in respective ctrl ASO mice. (D) Overview of duration of action study (n = 3 female mice/group, 6–9 weeks old mice, prior to disease onset). E-F. *Atp1a2* mRNA quantification at 2, 4, 8 and 12 weeks after CNS delivery of ASO1 (E) or ASO3 (F) represented as percentages of that in respective ctrl ASO mice. CX/MB, cortex/midbrain, CB/BS, cerebellum/brainstem, SC, spinal cord.

To determine an optimal *in vivo* working dose for the two lead ASOs from the *in vitro* screen, we next tested the potency and duration of target knockdown after a single bolus ICV administration of ASOs. We performed a dose response study using B6SJL WT mice treated with 25, 50, 75, 100, 400, or 700μg of ctrl ASO, and *Atp1a2* ASO1 or 3. Potent *Atp1a2* knockdown was achieved at doses as low as 50μg and 100μg for ASO1 and 50μg for ASO3 (S2A Fig) in WT mice. At higher doses of the ctrl ASO (400μg and 700μg), WT mice died spontaneously during suturing or in the recovery cage within 20mins post-ICV injection, thus, *Atp1a2* ASO administration at these higher doses were not performed. In a dose response study in the SOD1*G93A mice, ASO concentrations (100μg for ASO1 and 50μg for ASO3) that achieved *Atp1a2* knockdown in the WT animals led to lethality suggesting that WT mice had a higher tolerance of maximum dose of ASOs than age- and sex-matched SOD1*G93A mice (S2B Fig). Subsequently, we treated SOD1*G93A mice at 6–9 weeks of age, prior to disease onset, with a dose range of 25, 50, and 75μg of control, *Atp1a2* ASO1 or ASO3. Animals were sacrificed 2 weeks after ASO administration (Fig 3A). SOD1*G93A mice that received 75μg of ASO1 (n = 1/4), ASO3 (n = 3/3) and all mice that received 50μg of ASO3 (n = 3/3) died within 24h or during recovery within 30m after ICV-injection. Since mice treated with ctrl ASOs at similar concentrations recovered normally after ICV injection, we conclude that higher concentrations of *Atp1a2* ASOs cause toxicity *in vivo*. SOD1*G93A mice treated with 50μg of ASO1 (Fig 3B) and 25μg of ASO3 (Fig 3C) conferred ∼50–70% and ∼30–60% *Atp1a2* knockdown, respectively, 2 weeks after administration without overt toxicity, as defined by normal recovery and survival for 2 weeks after ICV ASO administration. Two weeks of ASO treatment did not cause changes in body weights in WT or SOD1*G93A animals (S2C and S2D Fig, respectively). Thus, to achieve at least 50% target reduction with minimal toxicity, we proceeded with two independent *Atp1a2* targeting ASOs, ASO1 at 50μg and ASO3 at 25μg, to characterize effects of *Atp1a2* knockdown in motor neuron disease in SOD1*G93A animals.

To measure the durability of *Atp1a2* knockdown in animals treated with a single bolus administration of ASOs, ASO1 (50μg), ASO3 (25μg), or associated ctrl ASOs were delivered via ICV into SOD1*G93A mice at 6–9 weeks of age, prior to disease onset, and mice were sacrificed at 2, 4, 8, or 12 weeks after ASO administration (Fig 3D). ASO1-treated mice maintained a ∼20–50% *Atp1a2* knockdown for 2–4 weeks and thereafter showed a further knockdown of ∼50–70% at 12 weeks after a single bolus administration (Fig 3E). Notably, ASO3 treatment induced a 40–60% knockdown from 2–4 weeks and a 60–70% knockdown of *Atp1a2* mRNA observed at 12 weeks following a single bolus administration (Fig 3F). A comparable extent of knockdown across different CNS regions was observed for both ASO1 and ASO3 (Fig 3E and 3F). We observed knockdown of α2-Na^+^/K^+^-ATPase protein in the spinal cord for 4 weeks (S3A Fig) after ASO1 treatment and this knockdown was sustained for as long as 12 weeks (S3C Fig; percentage α2-Na^+^/K^+^-ATPase protein knockdown at 4 weeks = 49%, 8 weeks = 43%, 12 weeks = 44%) consistent with the *Atp1a2* mRNA knockdown in the ASO1 treated SOD1*G93A mice. *Atp1a2* ASO3 treated SOD1*G93A mice exhibited 42% knockdown of α2-Na^+^/K^+^-ATPase protein in the spinal cord at 4 weeks after ASO treatment that is sustained, although to a lesser extent than in ASO1-treated mice, for as long as 12 weeks (percentage α2-Na^+^/K^+^-ATPase protein knockdown at 8 weeks = 35.4% and 12 weeks = 16.82%) (Figs 3A–3C and S3A–S3C). Collectively, these data show that a single bolus ICV administration of *Atp1a2* ASOs is sufficient to trigger sustained knockdown of *Atp1a2* mRNA in the CNS.

### Effect of *Atp1a2*-specific ASOs on disease onset and survival outcome in SOD1*G93A mouse model of familial ALS

**Fig 4:**
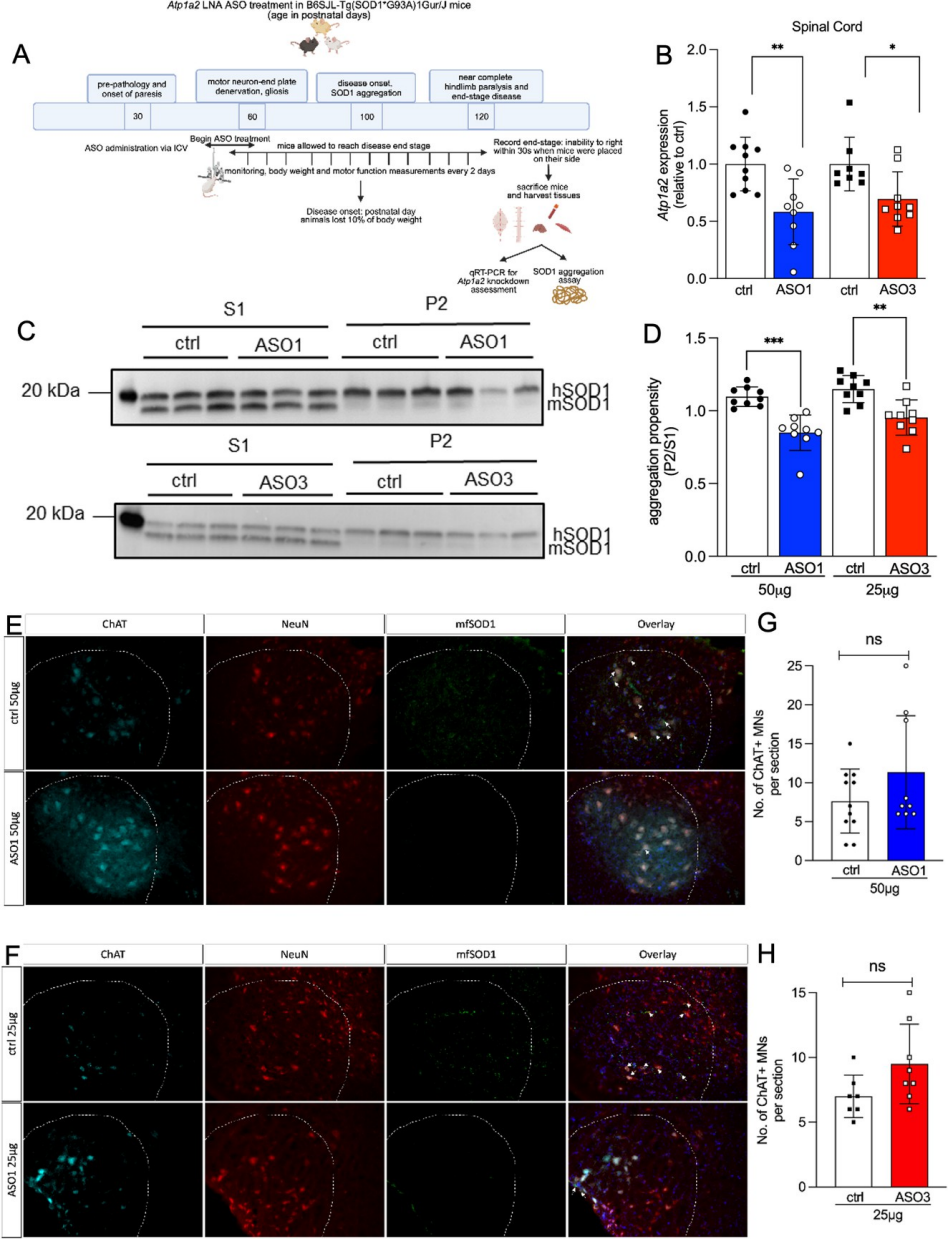
Reduced SOD1 aggregation in SOD1*G93A mice treated with *Atp1a2* ASOs without altering motor neuron loss. (A) Overview of experimental design. ASO1 (50μg, n = 23 mice:10 females, 13 males), ASO3 (25μg) or ctrl ASO (50 and 25μg; n = 21 mice: 10 females, 11 males in ASO3 and each control group) were delivered via ICV in 5–8 weeks old SOD1*G93A mice, prior to disease onset. (B) *Atp1a2* mRNA measured in spinal cords from end-stage SOD1*G93A mice treated with ASO1 (50μg), ASO3 (25μg) or ctrl ASO. Unpaired t-test with Welch’s correction, **p < 0.01, *p<0.05 (C). Representative immunoblots of detergent soluble and insoluble fractions of spinal cords from end-stage SOD1*G93A mice treated with single dose ICV injection of ASO1 (top panel) or ASO3 (bottom panel). (D) Quantification of relative aggregation propensity ‐ an index of the amount of SOD1 in the detergent-insoluble fraction (P2) compared to the detergent-soluble fraction (S1), n = 9 mice per group. Unpaired t-test with Welch’s correction, ***p<0.001, **p < 0.01. (E, F) Immunohistochemistry for motor neuron markers (NeuN, red and ChAT, cyan) and misfolded SOD1 (mfSOD1 C4F6, green) in spinal cord tissue from ctrl and ASO1- (E) or ASO3-treated (F) mice; DAPI-stained nuclei shown in blue in overlay images. (G, H) Quantification of ChAT+ motor neurons (MNs)/section in ASO1 v ctrl-treated mice in (G) and ASO3 v ctrl-treated mice in (H). Representative images at 20x magnification from n = 3 mice for ASO1 v ctrl and n = 2 mice for ASO3 v ctrl are shown in (E, F), scale bar represents 100μm and 3–4 sections per mouse were counted in a blinded manner, ns = not significant by unpaired t-test with Welch’s correction.

To evaluate whether lowering *Atp1a2* using ASOs might alter ALS disease course and outcome in SOD1*G93A mice, mice were treated with a single ICV bolus of either ctrl ASO (50 and 25μg), ASO1 (50μg), or ASO3 (25μg) at 5–8 weeks of age, prior to onset of phenotype of neurodegeneration (Fig 4A). Tissues were harvested from mice after animals reached disease end-stage. *Atp1a2* mRNA was significantly lowered in the spinal cord of end-stage SOD1*G93A mice treated with *Atp1a2*-specific ASO1 and ASO3 (Fig 4B). Expression of other alpha isoforms of the Na^+^/K^+^-ATPase pump, *Atp1a1* and *Atp1a3*, were comparable in ctrl and *Atp1a2* ASO-treated mice (S1B Fig), demonstrating *in vivo* specificity of the *Atp1a2* ASOs.

Aggregation of SOD1 in spinal cord tissue is a pathological hallmark in SOD1 mouse models [4] and post-morterm CNS tissues of familial ALS patients carrying SOD1 mutations [38, 39]. SOD1 aggregation, as defined by the formation of detergent-insoluble protein complexes, is present in the spinal cord of SOD1*G93A mice as early as postnatal day 30, at least 90 days before overt presentation of motor neuron pathology [40]. Thus, we assessed whether treatment with *Atp1a2*-specific ASOs impacts the abundance of SOD1 in spinal cord detergent insoluble fractions of end-stage SOD1*G93A mice. We subjected spinal cord tissue isolated from end-stage ctrl, *Atp1a2* ASO1 or ASO3-treated SOD1*G93A mice and age-matched controls to sequential detergent extraction and high speed centrifugation [5] to enrich for detergent insoluble protein aggregates. Spinal cord from SOD1*G93A mice treated with *Atp1a2*-specific ASOs exhibited significantly less detergent insoluble SOD1 aggregates, as detected using an antibody that recognizes total SOD1 protein, relative to age-matched ctrl ASO-treated animals (Figs 4C and 4D and S4). We next performed immunohistochemistry for misfolded SOD1 in spinal cord tissue from ctrl or *Atp1a2* ASO-treated SOD1*G93A mice following antigen retrieval and using an antibody that recognizes misfolded forms of mutant human SOD1 protein. We found that levels of misfolded SOD1 was reduced in *Atp1a2* ASO-treated mice compared with ctrl ASO (Fig 4E and 4F, white arrows). Further, immunohistochemical analyses of neuronal marker, NeuN and motor neuron marker, ChAT did not reveal motor neuron loss in response to *Atp1a2* ASO treatment (Fig 4E and 4F). On the contrary, we observed an enhanced intensity of ChAT staining in ASO1 and ASO3-treated mice however, no statistical significance was observed for the number of ChAT+ motor neurons between the ctrl and *Atp1a2* ASO-treated mice (Fig 4G and 4H). p62, a poly-ubiquitin binding protein and an autophagic receptor, accumulates in parallel and co-localizes with mutant SOD1 aggregates in the SOD1*G93A mouse spinal cord [41]. Levels of p62 remained consistent in spinal cord detergent insoluble fractions from mice receiving ctrl or *Atp1a2* ASO (S5 Fig). Thus, α2-Na^+^/K^+^-ATPase may play a specific role in regulating SOD1 aggregation.

**Fig 5:**
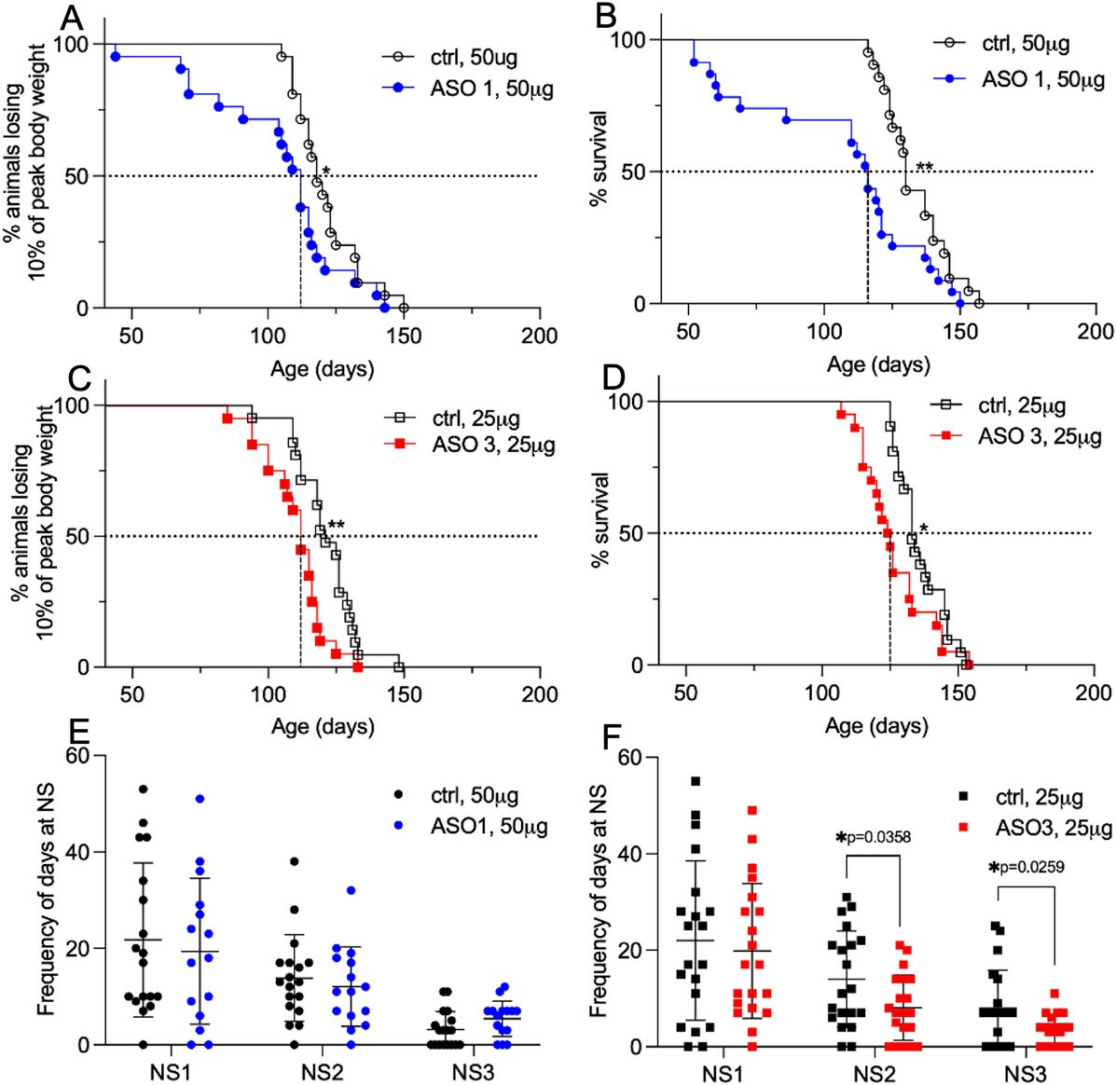
*Atp1a2* knockdown in SOD1*G93A mice results in accelerated disease onset and death with ASO3 treatment rescuing hindlimb motor function. Disease onset was defined as the age at which animals lose 10% of their peak body weight. (A) Median disease onset for ASO1-treated mice was 112 days compared with ctrl ASO-treated mice (118 days). (B) Median disease onset for ASO3-treated mice was 112 days compared with ctrl ASO-treated mice (121 days). Disease end-stage was defined as that point at which mice could not right themselves when placed on their side within 30s. (C) The median survival of SOD1*G93A mice treated with ASO1 was 116 days compared with ctrl ASO-treated mice (130 days). (D) The median survival of SOD1*G93A mice treated with ASO3 was 124.5 days compared with ctrl ASO-treated mice (133 days). Log-rank Mantel-Cox test, *p<0.05, **p<0.01. (E, F) Neurological scoring (NS) system adopted from ALS TDI to assess hindlimb function in SOD1*G93A mice- NS1: first symptoms (abnormal splay and/or tremble during tail suspension test), NS2: onset of paresis and NS3: paralysis. Frequency of days spent at NS stage for *Atp1a2* ASO1 and the ctrl ASO-treated mice (E, n = 15 mice) or *Atp1a2* ASO3 and the ctrl ASO-treated mice (F, n = 18 mice). n used for A-D as indicated in Fig 4A legend. Unpaired t-test with Welch’s correction, *p<0.05.

Despite marked reduction in *Atp1a2* mRNA and SOD1 aggregation, disease onset, defined as the postnatal day when mice lost 10% of their peak body weight, was significantly earlier in SOD1*G93A mice that underwent *Atp1a2* ASO treatment (ASO1 = 6 days (Fig 5A) and ASO3 = 9 days (Fig 5B)) compared with the ctrl ASO groups. Further, SOD1*G93A mice that received *Atp1a2* ASO1 had a median survival of 116 days, two weeks shorter than matched ctrl ASO mice (Fig 5C). Mice treated with *Atp1a2* ASO3 had a median survival of 124.5 days, a week shorter than ctrl ASO-treated mice (Fig 5D). Hindlimb deficits are the earliest signs of disease in SOD1*G93A mice. Therefore, we performed a blinded assessment of hindlimb function using the neurological scoring system (NeuroScore, NS, see methods). Briefly, NS0 indicates presymptompatic, NS1 represents onset of first symptoms such as abnormal splay or trembling during tail suspension, NS2 indicates onset of paresis, NS3 represents paralysis, and NS4 is disease endstage [26]. Mice exposed to *Atp1a2* ASO1 exhibited symptoms distributed across all the neurological scores, similar to ctrl ASO-treated mice (Fig 5E). Fewer *Atp1a2* ASO3 treated mice were scored with NS2 (onset of paresis) and NS3 (paralysis) compared with their respective ctrl ASO-treated mice (Fig 5F), suggesting the animals exhibited a less aggressive disease course. Collectively, these data suggest that *Atp1a2* ASO treatment led to earlier disease onset and death; however, the disease severity was more similar in ASO1 and milder in ASO3 treated mice compared to ctrl ASO-treated SOD1*G93A animals.

**Fig 6:**
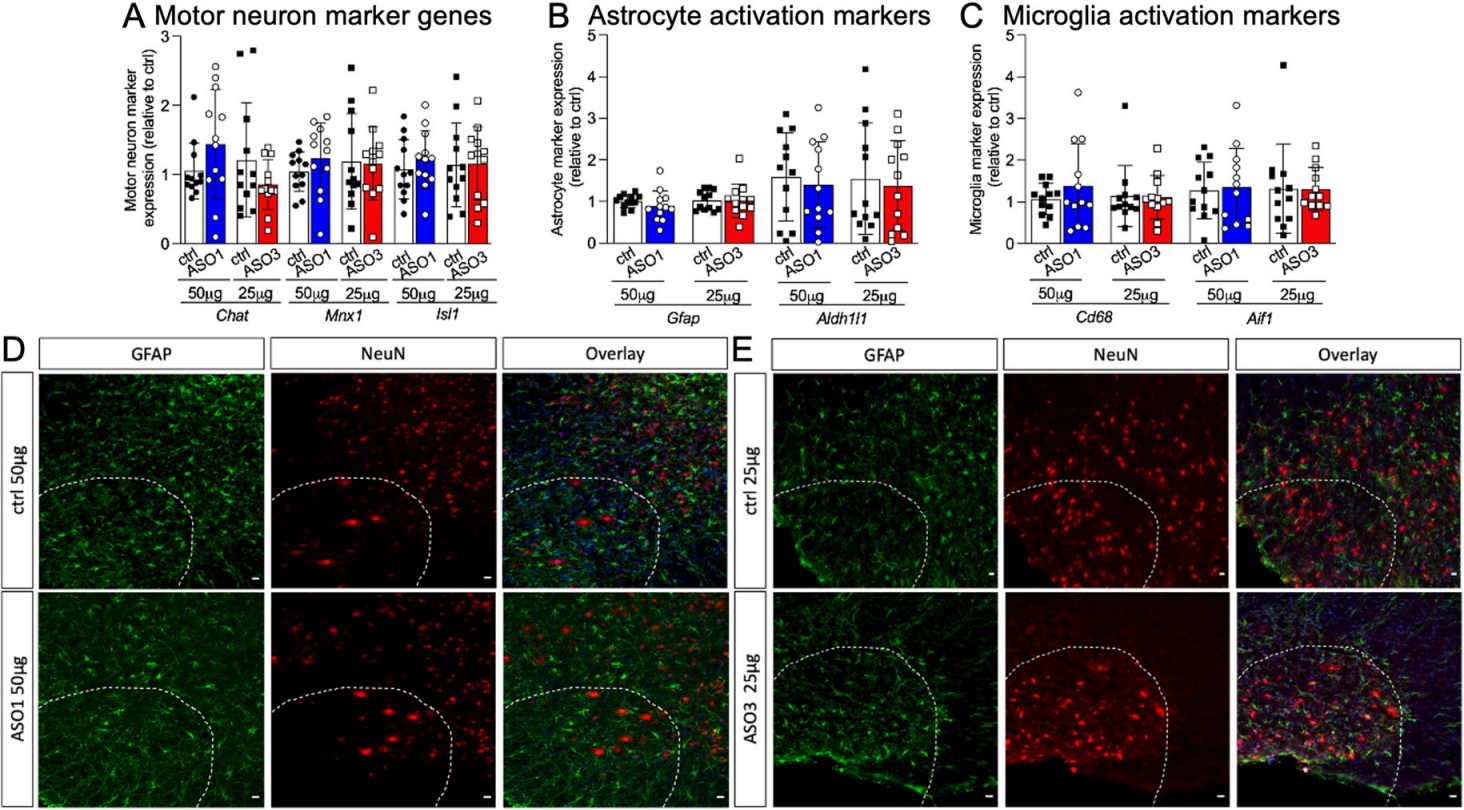
*Atp1a2* knockdown does not lead to motor neuron loss or alter classical astrocyte activation marker. Relative levels of transcripts for (A) motor neuron markers, (B) astrocyte, and (C) microglial reactivity in spinal cord lysates from a subset of SOD1*G93A mice shown in Fig 4 (n = 12 mice/group) as measured by qRT-PCR, molecular expression not significant between any groups by unpaired t-test with Welch’s correction. (D, E) Immunohistochemistry for motor neuron (NeuN, red) and astrocyte reactivity (GFAP, green) markers in ctrl, *Atp1a2* ASO1 (D) or ASO3 (E) treated SOD1*G93A spinal cord tissue; DAPI-stained nuclei are shown in blue in overlay images. One representative image from n = 3 mice for ASO1 v ctrl and n = 2 mice for ASO3 v ctrl-ASO treatment are shown in D and E.

Motor neuron death is most prominent in the spinal cord of end-stage SOD1*G93A mice [3]. To understand the molecular consequences of accelerated disease onset in *Atp1a2* ASO-treated SOD1*G93A mice, we measured spinal cord transcript levels of motor neuron marker genes (*Chat*, *Isl1*, *Mnx1*) by qPCR. Genes enriched in motor neurons were similar in ctrl and *Atp1a2* ASO-treated SOD1*G93A mice (Fig 6A). Markers of astrocyte (*Gfap* and *Aldh1l1*; Fig 6B) and microglial reactivity (*Cd68* and *Aif1*; Fig 6C) were also similar in spinal cord tissues from *Atp1a2* ASO and ctrl ASO-treated mice. Immunohistochemical (IHC) analyses of motor neurons (Fig 4E–4H: ChAT positive quantification per spinal cord section and Figs 4E, 4F, 6D and 6E: NeuN) and astrogliosis (GFAP, Fig 6D and 6E) were consistent with qPCR findings of similar gene expression levels of *Chat* and *Gfap* in ctrl and *Atp1a2* ASO-treated SOD1*G93A mice (Fig 6D and 6E). Thus, *Atp1a2* ASO treatment does not accelerate neuron loss or gliosis in SOD1*G93A mice.

### Absence of systemic toxicity in *Atp1a2* ASO treated SOD1*G93A mice

Next, we asked whether administration of *Atp1a2* ASOs in the CNS triggered systemic toxicity. To measure systemic effects of centrally administered ASOs, we compared *Atp1a2* levels in skeletal muscle, where the α2 isoform of Na^+^/K^+^-ATPase is highly expressed [42]. Intracereboventricular administration of *Atp1a2* specific ASOs had little or no effect on the levels of *Atp1a2* in the skeletal muscle of SOD1*G93A mice (S6 Fig). We also tested for signs of central and peripheral toxicity in response to the single bolus of ASOs administered in the CSF of SOD1*G93A mice. Purkinje cell layers in the cerebellum have been shown to be very sensitive to ASO treatment following central administration [43]. Therefore, we quantified mRNA levels of Purkinje cellular markers, *Calb1* and *Gad1*, in cerebellum lysates of *Atp1a2* ASO versus ctrl ASO-treated mice as a measure of toxicity to ASO treatment. Expression of these Purkinje cell markers was similar between the *Atp1a2* ASO and ctrl groups of mice (S7A and S7B Fig). Enzymes that signal liver damage or inflammation [44], alanine and aspartate aminotransferase, remained unchanged upon central administration of *Atp1a2* ASO and similar to that in ctrl ASO-treated SOD1*G93A mice (S7C and S7D Fig). Additionally, serum levels of creatinine (S7E Fig), a marker for kidney injury [45], were comparable in *Atp1a2* ASO or ctrl ASO-treated SOD1*G93A mice. Taken together, these data show that central administration of *Atp1a2* ASOs does not lead to overt central and systemic toxicity.

### ASO-mediated *Atp1a2* knockdown induces transcriptomic changes in primary mouse astrocytes and spinal cord tissue from SOD1*G93A mice

**Fig 7:**
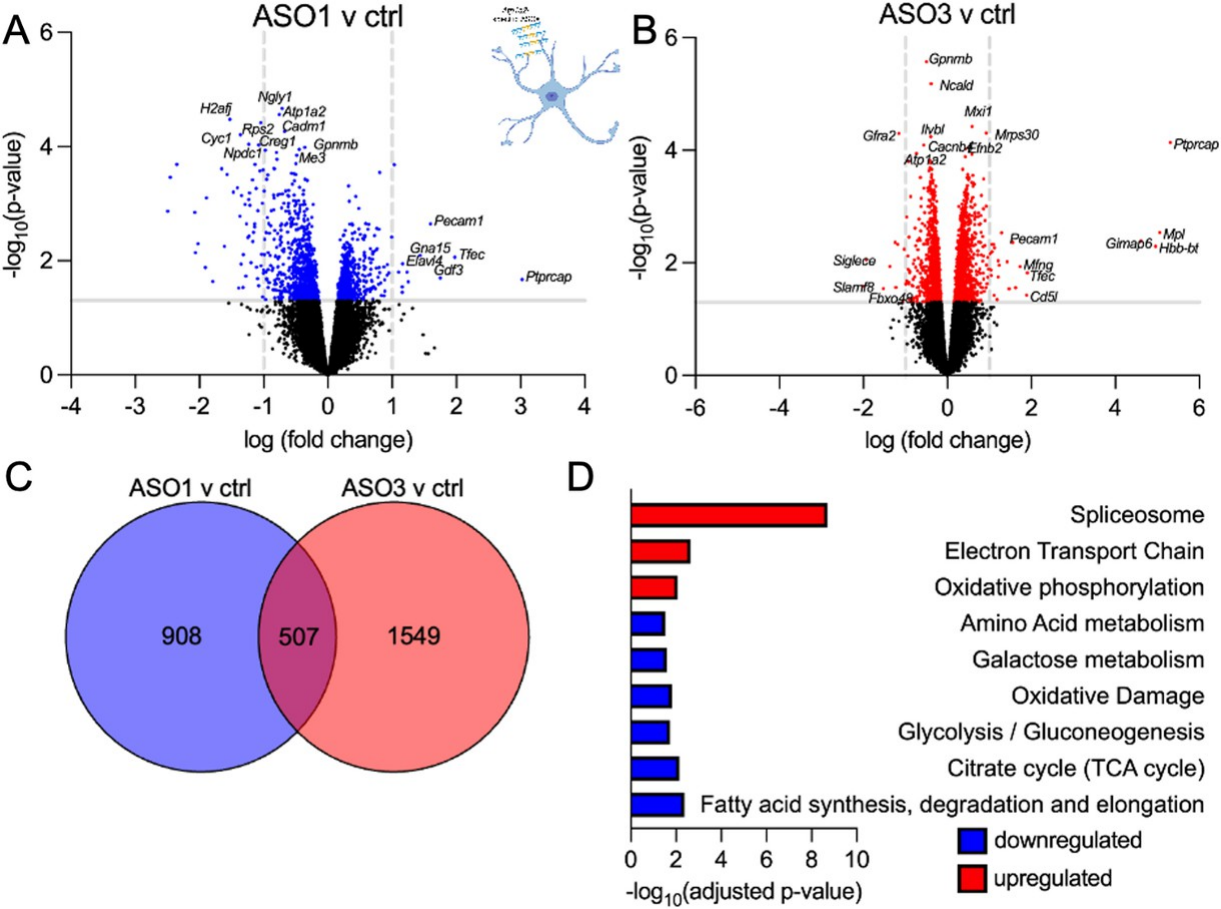
*Atp1a2* knockdown induces transcriptomic changes in primary mouse astrocytes from SOD1*G93A mice. Volcano plots showing differentially expressed genes between ASO1 (A) or ASO3 (B) vs ctrl ASO-treatment (total = 15085 variables, shown in blue and red are genes with p-value <0.05) in primary SOD1*G93A mouse astrocytes. (C) Venn diagram displaying the genes commonly altered in response to ASO1 and ASO3 treatment. (D) Pathway analyses of the differentially expressed genes shared between ASO1 and ASO3.

Cre-mediated selective gene excision of mutant SOD1 in astrocytes from birth significantly delays disease onset and/or progression via an indirect effect on microglial activation in transgenic mice expressing two distinct familial ALS-linked SOD1 mutations [46, 47]. Conversely, expression of SOD1*G93A in astrocytes alone is sufficient to induce motor neuron death and dysfunction in an *in vivo* study involving transplantation of glial precursor cells harboring SOD1*G93A into cervical spinal cords of WT rats [48]. This finding builds on prior work supporting that astrocyte cell-intrinsic pathways actively contribute to motor neuron degeneration and that disease onset is non-cell autonomous [49]. To define astrocyte cell-intrinsic effects after *Atp1a2* knockdown, we generated primary mouse astrocytes from SOD1*G93A P2 pups and nucleofected them with ctrl ASO or *Atp1a2*-specific ASO1 or ASO3. Transcriptome-wide analyses revealed 1415 differentially expressed genes (p<0.05: 535 upregulated and 880 downregulated genes) when comparing *Atp1a2-*specific ASO1 to ctrl ASO (Fig 7A and S3 Table) and 2056 differentially expressed genes (p<0.05: 1080 upregulated and 976 downregulated genes) when comparing *Atp1a2-*specific ASO3 to ctrl ASO (Fig 7B and S4 Table). We focused on those genes that were differentially expressed in ASO1 and ASO3-treated astrocytes to prioritize common genes downstream of *Atp1a2* knockdown (Fig 7C and S5 Table). Using transcriptomics, mouse astrocytes have been shown to adopt a neurotoxic ‘A1’ phenotype upon exposure to LPS activated microglia-derived IL-1⍺, TNF and C1q and alternately, they can adopt a neuroprotective ‘A2’ phenotype in ischemic stroke; both of which are acute pathological states [50, 51]. *Atp1a2* knockdown in SOD1*G93A astrocytes led to upregulation of A2-reactive genes (*Ptx3* and *S100a10)*, which are associated with negative outcomes in ALS [50, 52, 53]. *S100a6*, a gene encoding a Ca^2+^/Zn^2+^ binding protein, was elevated in *Atp1a2* ASO-treated astrocytes and in SOD1*G93A mice and sporadic ALS patients (S8 Fig and S6 Table) [54]. Additionally, *Atp1a2* knockdown led to downregulation of the neurotoxic A1-reactive, oxidative response gene (*Ugt1a7c*), and genes downregulated in aging astrocytes (*Prom1* and *Tnc)* [55, 56]. Genes upregulated in response to ASO-mediated *Atp1a2* knockdown were involved in RNA processing, degradation and transport, cytosolic ribosomal proteins, mitochondrial electron transport chain, and oxidative phosphorylation whereas downregulated genes were involved in metabolic pathways (fatty acid, glucose/pyruvate/TCA cycle, amino acid) (Fig 7D and S7 Table). Thus, these findings suggest that the silencing of *Atp1a2* may lead to altered metabolic pathways in astrocytes and impairs their ability to mount protective responses against oxidative stress.

**Fig 8:**
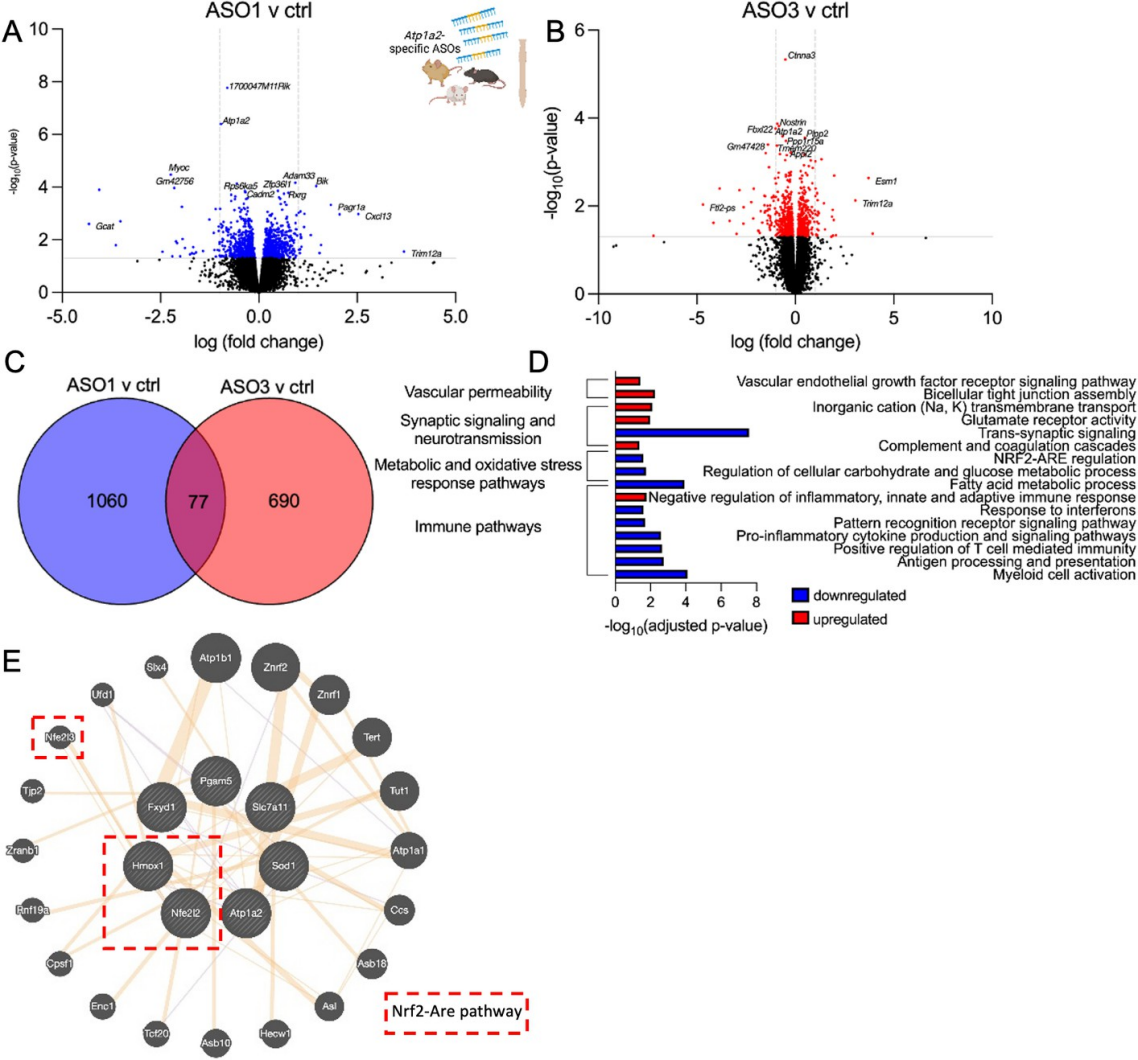
*Atp1a2* knockdown induced transcriptomic changes in spinal cord tissue from SOD1*G93A mice. Volcano plots showing differentially expressed genes between ASO1 (A) or ASO3 (B) vs ctrl ASO-treated spinal cord tissue (shown in blue and red are genes with p-value <0.05). (C) Venn diagram displaying the genes commonly altered in response to ASO1 and ASO3 treatment. (D) Pathway analyses of the differentially expressed genes shared between ASO1 and ASO3. (E) GeneMANIA network of Nrf2-ARE pathway genes (dotted red box) with *Atp1a2*, *Fxyd1 and Sod1* altered in response to *Atp1a2* ASO treatment.

To determine whether *Atp1a2* knockdown impacts molecular pathways beyond astrocytes, transcriptomic analyses were performed in total RNA extracted from spinal cord tissue. We identified 1140 differentially expressed genes (p<0.05: 581 upregulated and 559 downregulated genes) when comparing *Atp1a2* ASO1 to ctrl ASO (Fig 8A and S8 Table) and 768 differentially expressed genes (p<0.05: 339 upregulated and 429 downregulated genes) when comparing *Atp1a2* ASO3 to ctrl ASO (Fig 8B and S9 Table). We identified 77 differentially expressed genes differentially expressed between both *Atp1a2-*ASO treatments (Fig 8C and S10 Table). Of these commonly differentially expressed genes, genes upregulated in response to *Atp1a2* knockdown were enriched for pathways associated with glutamate receptor signaling, post-synaptic neurotransmitter receptor activity and localization to synapse, ion channel activity, negative regulation of innate and adaptive immune systems, activation of complement cascades leading to the formation of membrane attack complexes, coagulation, and increased permeability of the blood-brain-barrier (Fig 8D and S11 Table). Genes downregulated in response to *Atp1a2* knockdown were associated with pathways involving trans-synaptic signaling, vesicle-mediated transport in synapse, innate immune activation, antigen presentation, and adaptive immune responses, pattern recognition receptor signaling, proinflammatory cytokine production and NF-κB signaling, chemokine production and receptor activity, response to interferons, cell death and senescence, and metabolic pathways (Fig 8D and S12 Table). Genes encoding oxidative and other stress response pathways were also downregulated upon *Atp1a2* knockdown (Fig 8D and S12 Table) in the spinal cord. Collectively, transcriptomics data reveal downregulation of metabolic pathways to be a common element in *Atp1a2* ASO-treated SOD1*G93A primary astrocytes and spinal cord. However, upregulation of glutamate receptor signaling, complement cascade activation and downregulation of trans-synaptic signaling, oxidative response, and immune activation pathways were only observed in spinal cord tissue from *Atp1a2* ASO-treated mice. Thus, transcriptomics studies highlight the non-cell autonomous impact of altering astrocyte specific *Atp1a2* in this motor neuron permissive environment.

## Discussion

Astrocyte-specific upregulation of the ion pump α2-Na^+^/K^+^-ATPase triggers motor neuron death in ALS suggesting its suitability as a promising therapeutic target [9]. In this study, we tested the hypothesis that α2-Na^+^/K^+^-ATPase downregulation using ASOs might ameliorate disease course in SOD1*G93A mice. We have found two ASOs targeting *Atp1a2* that when administered before disease onset cause marked downregulation of *Atp1a2* and substantial reduction of SOD1 aggregation in the spinal cord of SOD1*G93A mice. *Atp1a2* ASO3 treated SOD1*G93A mice also exhibit less aggressive disease at the onset of paresis and paralysis stages as compared to control mice. However, *Atp1a2*-specific ASO treatment leads to earlier disease onset and reduced survival than control ASO-treated SOD1*G93A mice. Notably, SOD1*G93A mice treated with *Atp1a2* ASOs do not show signs of systemic toxicity. Transcriptomic studies reveal that *Atp1a2* knockdown using ASOs causes perturbations in key cellular processes, metabolic pathways, and markers of astrocyte activation defined in acute pathological contexts in primary SOD1*G93A astrocytes whereas ASO treatment in SOD1*G93A mice impacts oxidative stress response, immune activation, trans-synaptic and glutamatergic signaling, and the complement pathway in the spinal cord. Our results uncouple SOD1 aggregation and disease course and show that downregulation of *Atp1a2* by ASOs before disease onset fails to slow motor neuron disease in mutant SOD1 mice. This phenomenon observed in our study is consistent with prior studies in other neurodegenerative diseases [57–59], and a recent report on soluble misfolded SOD1 being the disease driver in ALS disease [60]. Overall, these observations contrast with the finding of significant delay in disease onset and extension of lifespan (19.5 ± 2.6 days), together with improved motor function in SOD1*G93A mice heterozygous for *Atp1a2* from birth [9]. Thus, the timing of *Atp1a2* modulation may be critical to mitigate astrocytic α2-Na^+^/K^+^-ATPase driven non-cell autonomous neurodegeneration in ALS.

SOD1 aggregation is thought to be a major contributor to mutant-mediated toxicity and ALS disease progression [4, 38–40, 61]. Interestingly, direct interaction of all isoforms of Na^+^/K^+^-ATPases with mutant SOD1 has been demonstrated in spinal cord proteomic analysis from symptomatic mutant SOD1 transgenic mice [62]. Taken together with our finding of reduced SOD1 aggregation in *Atp1a2* ASO-treated mice, this suggests a potential role for Na^+^/K^+^-ATPase in SOD1 aggregation. *Atp1a2* and SOD1 are co-expressed in GeneMania network analyses of transcriptomics data of *At1pa2* and control ASO-treated mice (Fig 8E). Furthermore, transcriptomics studies in these mice also show upregulation of the lysosomal degradative enzyme, *Ctsc* (S13 Table), suggesting a possible explanation for reduced SOD1 aggregates upon *Atp1a2* knockdown.

LNA-modified ASOs show stronger knockdown than other gapmer modifications, though LNA ASOs may lead to toxicity, in these studies, LNA ASOs were administered via subcutaneous or intraperitoneal routes [63–65]. Studies of CNS delivery of LNA-modified ASO gapmers are limited; however, little or no toxicity of LNA ASOs in the rat brain has been observed in some studies [66]. In our study, CSF delivery of *Atp1a2*-specific LNA ASOs did not cause liver or kidney toxicity, and *Atp1a2* expression was unaffected in the skeletal muscle in treated SOD1*G93A mice. Transcriptomics studies showed that *Atp1a2* ASOs downregulated in mice innate immune genes encoding pattern receptor recognition (involving *Tlr3*, *Tlr7*, *and Tlr9*), interferon, and adaptive immune activation pathways (S12 Table). There was also downregulation of the NOD-like receptor (NLR) signaling, IL-1β production and signaling pathway, IL-18 production, and NF-κB signaling in spinal cord tissue from *Atp1a2* ASO-treated SOD1*G93A mice (S12 Table). Together, these observations suggest the immune pathways may not be activated in response to *Atp1a2* ASO treatment (Fig 8D and S11 and S12 Tables). Interestingly, cellular senescence and cell death pathways (programmed cell death, necroptosis, and apoptosis) were also downregulated in response to *Atp1a2* knockdown (Fig 8D and S12 Table). Nonetheless, a larger screening effort might be required to identify ASOs targeting *Atp1a2* with lower on-target-induced adverse effects.

**Fig 9:**
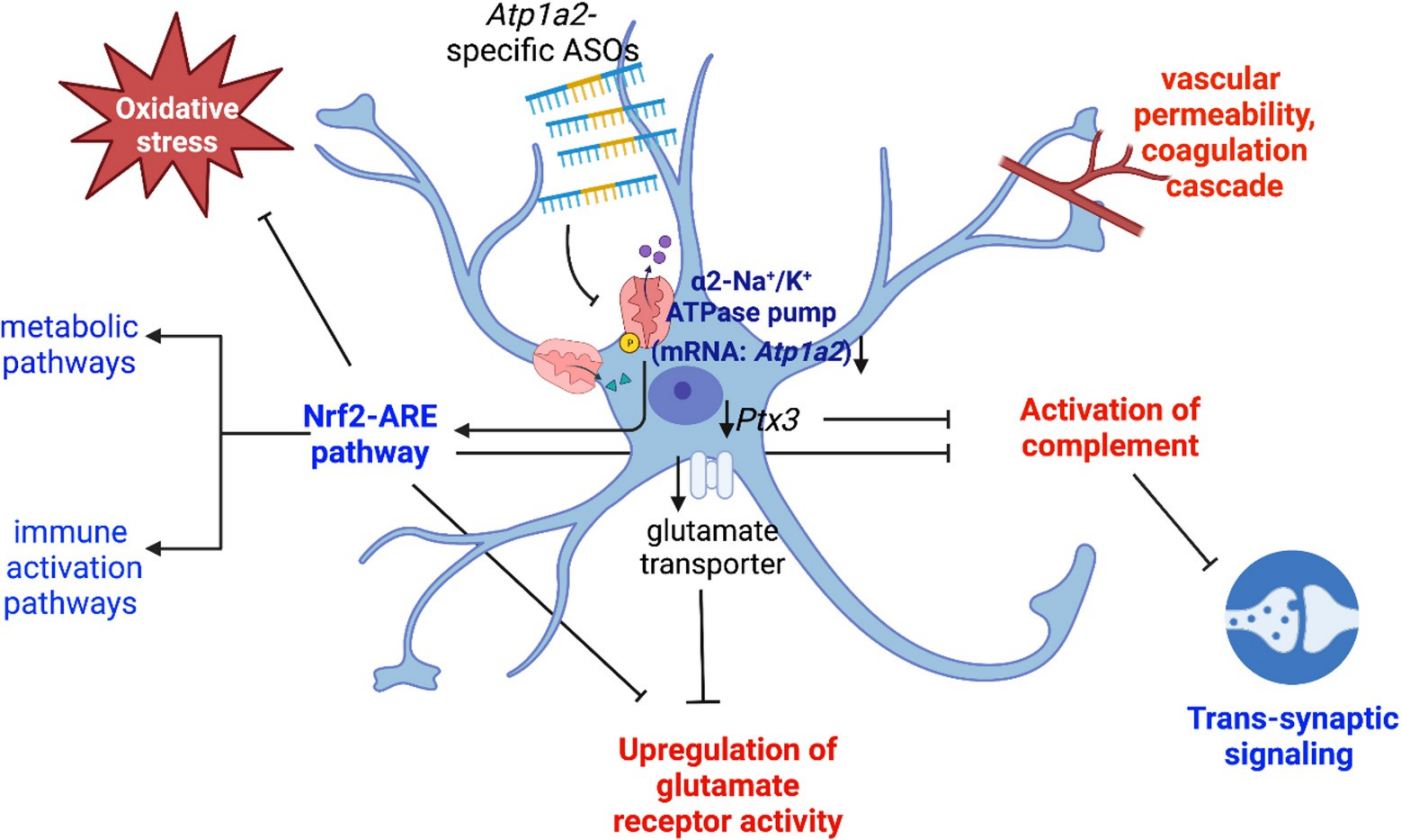
Summary of transcriptomic changes in response to *Atp1a2* ASO treatment. Knockdown of *Atp1a2* specifically expressed in astrocytes by ASOs leads to downregulation (highlighted as blue font) of the oxidative stress response Nrf2-ARE, metabolic, immune activation and trans-synaptic signaling pathways. Genes upregulated (highlighted as red font) in response to *Atp1a2* knockdown fall into glutamate receptor activity, activation of complement and coagulation cascades and vascular permeability. Blunt arrows indicate inhibition, sharp arrows indicate activation, and downwards arrow indicates downregulation.

Transcriptomic studies in the spinal cord tissue suggest a few possible mechanisms for the observed outcomes in the *Atp1a2* ASO-treated SOD1*G93A mice. We observed downregulation of the oxidative stress-responsive Nrf2-ARE pathway components ‐ *Hmox1*, *Pgam5*, *Slca711* in our RNA-seq dataset from *Atp1a2* ASO-treated spinal cord (S12 Table). Oxidative stress and reactive oxygen species are well-researched contributors to ALS pathogenesis, and small molecule modulators to promote the Nrf2 and Hmox1 signaling pathway are being explored in ALS and other neurological diseases [67, 68]. Interestingly, Nrf2 has also been shown to be a direct target of α1-Na^+^/K^+^-ATPase in human adenocarcinoma cells [69]. These data suggest direct crosstalk between *Atp1a2* and the Nrf2-ARE pathway (Fig 8E). A majority of genes that are downregulated in response to *Atp1a2* knockdown in primary SOD1*G93A astrocytes and the spinal cord fall into key metabolic pathways central to astrocyte-neuron crosstalk [70, 71], such as glycolysis, gluconeogenesis, pyruvate and central carbon metabolism, citrate cycle, and fatty acid oxidation. We also observe an upregulation of genes involved in the mitochondrial electron transport chain and oxidative phosphorylation upon ASO-mediated *Atp1a2* knockdown in SOD1*G93A astrocytes. Upregulation of oxidative phosphorylation and downregulation of carbohydrate and glycolytic pathways were also observed in spinal cord tissue from *Atp1a2* ASO-treated SOD1*G93A mice. The Nrf2-ARE oxidative stress response pathway which we found to be downregulated in *Atp1a2* ASO-treated mice has been shown in other studies to modulate cytokine signaling and metabolic pathways [67], thereby, implying a possible central role for the Nrf2-ARE pathway in the downregulation of these pathways upon *Atp1a2* knockdown (Fig 9).

In a mouse model of Alzheimer’s disease, deficits in synaptic plasticity and memory due to astrocytic glycolysis impairment early in the disease have been reported [72]. Consistent with the downregulation of the glycolytic pathway in *Atp1a2* ASO-treated SOD1*G93A mice, we found downregulation of trans-synaptic signaling and vesicle-mediated transport in the synapse. The downregulation of synaptic signaling could also be attributed to the upregulation of complement activation pathways in these mice possibly due to excessive complement-mediated pruning [73]. Other genes upregulated in *Atp1a2* ASO-treated mice are involved in glutamate receptor and ion channel activity (Ca^2+^, Na^+^, and K^+^). Astrocytes have been previously shown to regulate glutamate uptake from the extracellular space through modulation of α2-Na^+^/K^+^-ATPase [10, 74] and in our study, we found downregulation of the predominant glutamate transporters in response to *Atp1a2* knockdown (S13 Table), thereby contributing to the observed upregulation of glutamate receptor activity (Fig 9).

Overall, our RNA-seq findings highlight *Atp1a2* knockdown-induced downregulation of the Nrf2-ARE oxidative stress response pathway and downstream effects on astrocyte metabolic and immune activation pathways, altered astrocyte reactivity state resulting in complement activation and reduced trans-synaptic signaling owing to excessive pruning, reduced expression of glutamate transporter and concomitant upregulation of glutamate receptor activity as the three potential mechanisms responsible for earlier disease onset and poorer survival outcome in *Atp1a2* ASO-treated mice despite causing a reduction in SOD1 aggregation (Fig 9).

An important question that remains to be addressed is the discrepancy in the outcome between *Atp1a2* ASO treatment in SOD1*G93A mice before disease onset in our current study, and the intraspinal delivery of lentivirus-encoded *Atp1a2* siRNA at post-natal day 90 [9], which corresponds with disease onset in the SOD1*G93A mice [75]. Although *Atp1a2* ASO treatment did not affect motor neuron death, lentivirus-encoded *Atp1a2* siRNA promotes motor neuron survival without an impact on astrocyte or microglial abundance [9]. The difference in the ASO vs lentivirus-encoded siRNA approach to achieve *Atp1a2* knockdown together with the timing of intervention and delivery route of agent administration might be potential reasons for the observed disparity in motor neuron survival outcome. Taken together, these observations also suggest a disease stage-specific role for α2-Na^+^/K^+^-ATPase in ALS. Although ASOs and siRNAs are developed as gene-silencing therapeutics, thus far ASOs demonstrate wider tissue and cellular distribution in the CNS than siRNAs from the initial site of infusion [43, 76, 77]. Thus, addressing the limitations of our current study namely, identifying the correct temporal window to knockdown α2-Na^+^/K^+^-ATPase might aid in the development of α2-Na^+^/K^+^-ATPase-based therapeutics for ALS. Furthermore, crossing astrocyte-specific conditional α2-Na^+^/K^+^-ATPase knockout mice [78] with SOD1*G93A mice might provide a better system to understand the disease stage-specific role of α2-Na^+^/K^+^-ATPase in ALS. Because α2-Na^+^/K^+^-ATPase is upregulated in both familial and sporadic ALS patients [9], α2-Na^+^/K^+^-ATPase remains a potential therapeutic target for ALS; however, further work is required to understand the temporal role, regulation, and therapeutic modulation of α2-Na^+^/K^+^-ATPase in ALS.

## Supporting information

S1 Fig *Atp1a2* ASOs specifically target *Atp1a2*. (A) Relative levels of *Atp1a1* mRNA in primary mouse astrocytes (WT), 48h after nucleofection with *Atp1a2* ASO1, ASO3 or ctrl ASO. Shown here are pooled fold change values from 3 independent experiments, ns = not significant by one-way ANOVA. (B) *Atp1a1* and *Atp1a3* mRNA levels in spinal cord lysates from a subset of SOD1*G93A mice shown in Fig 4 (n = 12 mice/group), molecular expression not significant by unpaired t-test with Welch’s correction.
(TIF)

S2 Fig Dose response study in WT and SOD1*G93A mice. (A) Relative levels of *Atp1a2* mRNA in different central nervous system regions of WT mice, 2 weeks after ICV injection with *Atp1a2* ASO1, ASO3 or ctrl ASO at indicated concentrations. (B) Higher maximum tolerated dose of LNA ASOs observed in WT than in SOD1*G93A mice. (C, D) Body weights of WT and SOD1*G93A mice treated with PBS, ctrl or *Atp1a2* ASO1 or ASO3 before and two weeks post-ICV injection (n = 4 mice/group).
(TIF)

S3 Fig α2-Na+/K+-ATPase knockdown in spinal cord from ctrl, *Atp1a2* ASO1 or ASO3-treated SOD1*G93A mice. Immunoblot images of α2-Na+/K+-ATPase protein levels in ctrl, *Atp1a2* ASO1 or ASO3-treated mouse spinal cord (top lane) and housekeeping protein GAPDH (bottom lane) at (A) 4 weeks, (B) 8 weeks, or (C) 12 weeks post-ASO treatment.
(TIF)

S4 Fig PonceauS staining to demonstrate equal protein loading, in S1 fractions from spinal cord homogenates used for SOD1 aggregation assay immunoblot images shown in Fig 4C. (TIF)

S5 Fig Insoluble p62 is not altered by *Atp1a2* knockdown in SOD1*G93A mice. (A) Representative immunoblots and (B) quantitation of the amount of p62 in detergent soluble (S1) and insoluble fractions (P2) of spinal cords from end-stage SOD1*G93A mice treated with single dose ICV injection of ctrl ASO, *Atp1a2* ASO1 (A, top panel) or ASO3 (A, bottom panel). N = 9 mice per group. ns = not significant by unpaired t-test with Welch’s correction.
(TIF)

S6 Fig CNS administered *Atp1a2*-specific ASOs do not affect *Atp1a2* expression in skeletal muscle. Relative *Atp1a2* transcript levels in skeletal muscle (n = 5 mice/group from a subset of SOD1*G93A mice shown in Fig 4, molecular expression not significant by unpaired t-test with Welch’s correction.
(TIF)

S7 Fig *Atp1a2*-specific ASOs fail to cause central or systemic toxicity in treated SOD1*G93A mice. Purkinje cell markers, (A) *Calb1* and (B) *Gad1* in cerebellum lysates from a subset of SOD1*G93A mice shown in Figs 4 and 5 (n = 8–10 mice/group) were measured by qRT-PCR. Serum measurements of (C) alanine aminotransferase (ALT), (D) aspartate aminotransferase (AST) enzymes and creatinine (E), for signs of liver and kidney toxicity respectively, in a subset of end-stage SOD1*G93A mice shown in Fig 4 (n = 5–7 mice/group). Molecular expression (A, B) and serum levels of markers (C, D, E) not significant by unpaired t-test with Welch’s correction.
(TIF)

S8 Fig Overlap of genes differentially expressed in response to *Atp1a2* ASO treated primary SOD1*G93A mouse astrocytes and published astrocyte datasets. Heat map of genes impacted by ASO treatment and intersecting with astrocyte activation, reactivity, and aging.
(TIF)

S1 Raw images Original images for immunoblotting experiments in main and supporting figures. (PDF)

S1 Table List of PCR primers used in this study. (XLSX)

S2 Table Sequence of LNA ASOs used in this study. Five different LNA ASOs as listed are single stranded oligonucleotides about 15–16 nucleotides long, enriched with LNA in the flanking regions with a central gap of unmodified DNA, hence referred to as GapmeRs. The antisense LNA GapmeRs have phosphorothioate backbone modifications as indicated by ‘*’ in the sequence. Negative control A (ctrl), *Atp1a2*-specific ASO1 and ASO3 were purchased for both *in vitro* and *in vivo* experiments and the corresponding catalog numbers are listed in the top and bottom sub-rows, respectively.
(XLSX)

S3 Table Differentially expressed gene list for *Atp1a2* ASO1 v ctrl ASO-treated SOD1*G93A primary mouse astrocytes. p<0.05: 535 upregulated and 880 downregulated genes corresponding to the volcano plot in Fig 7A.
(XLSX)

S4 Table Differentially expressed gene list for *Atp1a2* ASO3 v ctrl ASO-treated SOD1*G93A primary mouse astrocytes. p<0.05: 1080 upregulated and 976 downregulated genes corresponding to the volcano plot in Fig 7B.
(XLSX)

S5 Table List of overlapping genes between *Atp1a2* ASO1 and ASO3 treatment in SOD1*G93A primary mouse astrocytes. 507 commonly differentially expressed genes between *Atp1a2* ASO1 and ASO3 treatments as shown in Fig 7C.
(XLSX)

S6 Table Log fold change and p-values for listed genes in *Atp1a2* ASO1 and ASO3 treated SOD1*G93A primary mouse astrocytes. Corresponding to overlap with published datasets for mouse astrocytes as displayed in S8 Fig.
(XLSX)

S7 Table Pathway analyses for upregulated and downregulated genes in *Atp1a2* ASO1 and ASO3 treated SOD1*G93A primary mouse astrocytes, corresponding to pathways depicted in Fig 7D. (XLSX)

S8 Table Differentially expressed gene list in spinal cord from *Atp1a2* ASO1 v ctrl ASO-treated SOD1*G93A mice. p<0.05: 581 upregulated and 559 downregulated genes corresponding to the volcano plot in Fig 8A.
(XLSX)

S9 Table Differentially expressed gene list in spinal cord from *Atp1a2* ASO3 v ctrl ASO-treated SOD1*G93A mice. p<0.05: 339 upregulated and 429 downregulated genes corresponding to the volcano plot in Fig 8B.
(XLSX)

S10 Table List of overlapping genes in spinal cord from *Atp1a2* ASO1 and ASO3 treated SOD1*G93A mice. 77 commonly differentially expressed genes between *Atp1a2* ASO1 and ASO3 treatments as shown in Fig 8C.
(XLSX)

S11 Table Pathway analyses for upregulated genes in spinal cord from *Atp1a2* ASO1 and ASO3 treated SOD1*G93A mice, corresponding to pathways depicted in Fig 8D. (XLSX)

S12 Table Pathway analyses for downregulated genes in spinal cord from *Atp1a2* ASO1 and ASO3 treated SOD1*G93A mice, corresponding to pathways depicted in Fig 8D. (XLSX)

S13 Table Lysosomal, glutamate transporter, RNaseh1 and Cdkn1a genes in response to Atp1a2 ASO treatment in SOD1*G93A mouse spinal cord. (XLSX)
