# Supplementary figures and images for: Targeted ASO-mediated Atp1a2 knockdown in astrocytes reduces SOD1 aggregation and accelerates disease onset in mutant SOD1 mice
Source: PLoS One. 2023 Nov 28;18(11):e0294731. doi: 10.1371/journal.pone.0294731 (PMC10683999; doi:10.1371/journal.pone.0294731)

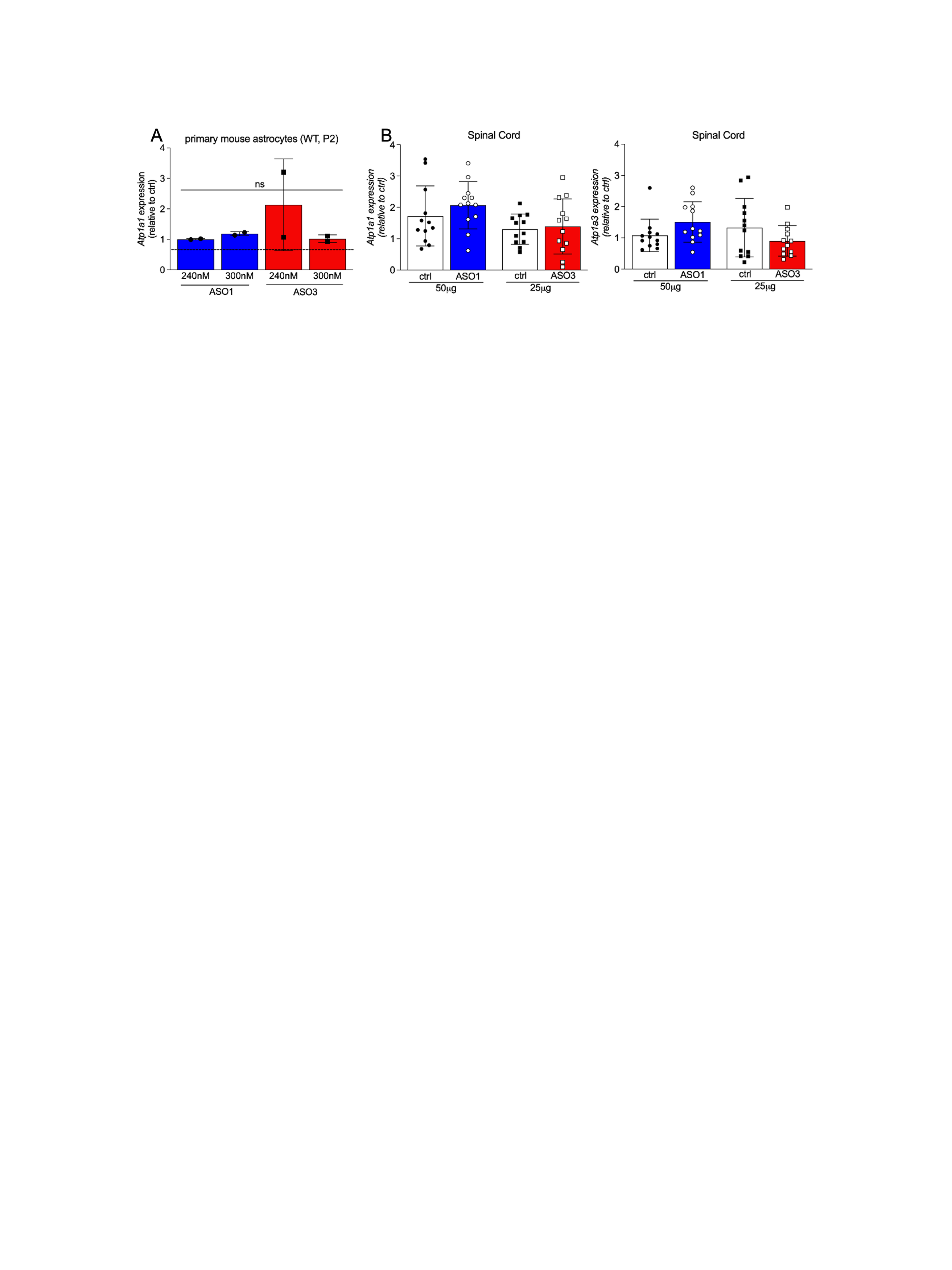

Supplement: S1 Fig — (A) Relative levels of Atp1a1 mRNA in primary mouse astrocytes (WT), 48h after nucleofection with Atp1a2 ASO1, ASO3 or ctrl ASO. Shown here are pooled fold change values from 3 independent experiments, ns = not significant by one-way ANOVA. (B) Atp1a1 and Atp1a3 mRNA levels in spinal cord lysates from a subset of SOD1*G93A mice shown in Fig 4 (n = 12 mice/group), molecular expression not significant by unpaired t-test with Welch’s correction. (TIF) [file pone.0294731.s001.tif]

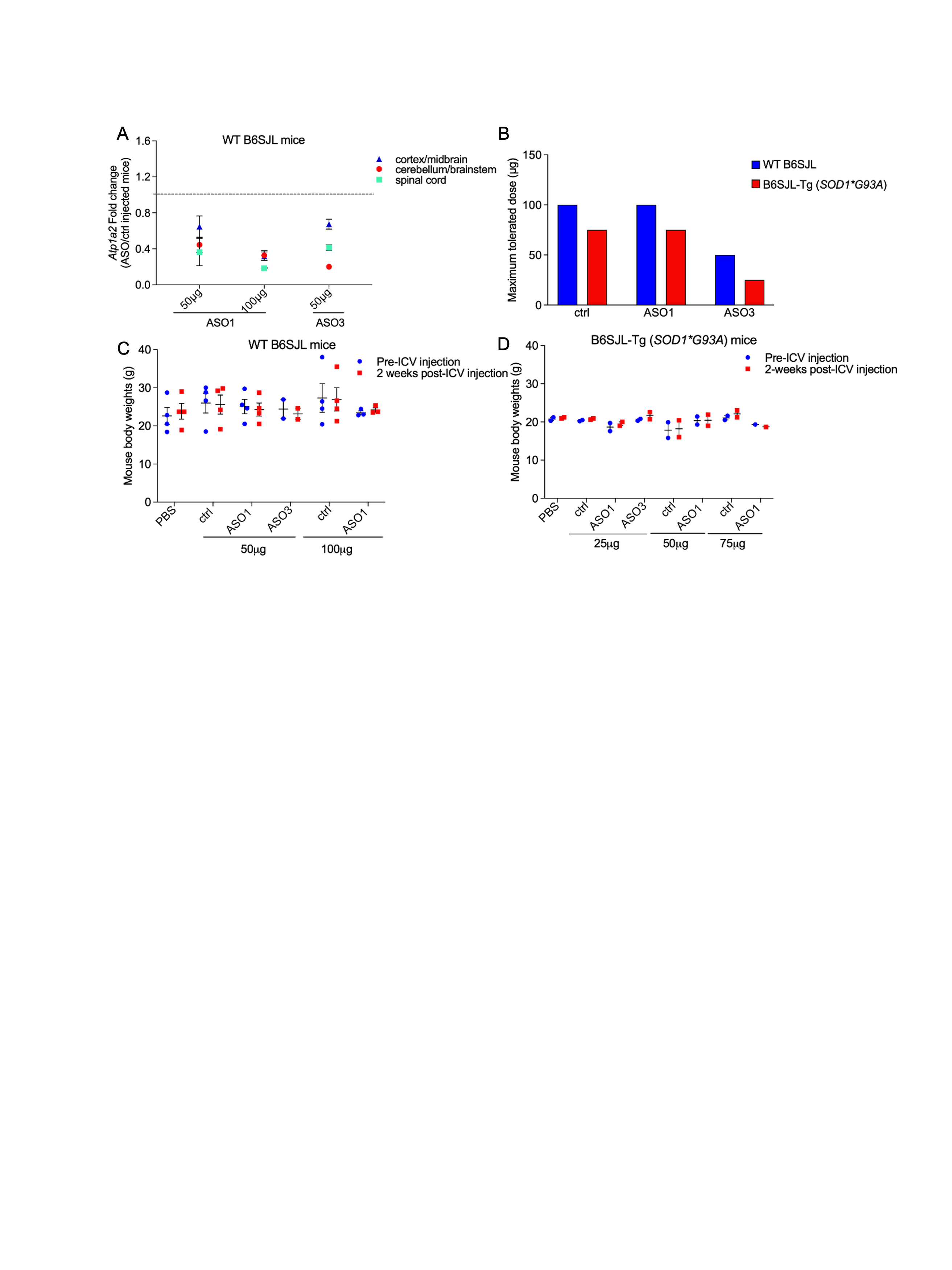

Supplement: S2 Fig — (A) Relative levels of Atp1a2 mRNA in different central nervous system regions of WT mice, 2 weeks after ICV injection with Atp1a2 ASO1, ASO3 or ctrl ASO at indicated concentrations. (B) Higher maximum tolerated dose of LNA ASOs observed in WT than in SOD1*G93A mice. (C, D) Body weights of WT and SOD1*G93A mice treated with PBS, ctrl or Atp1a2 ASO1 or ASO3 before and two weeks post-ICV injection (n = 4 mice/group). (TIF) [file pone.0294731.s002.tif]

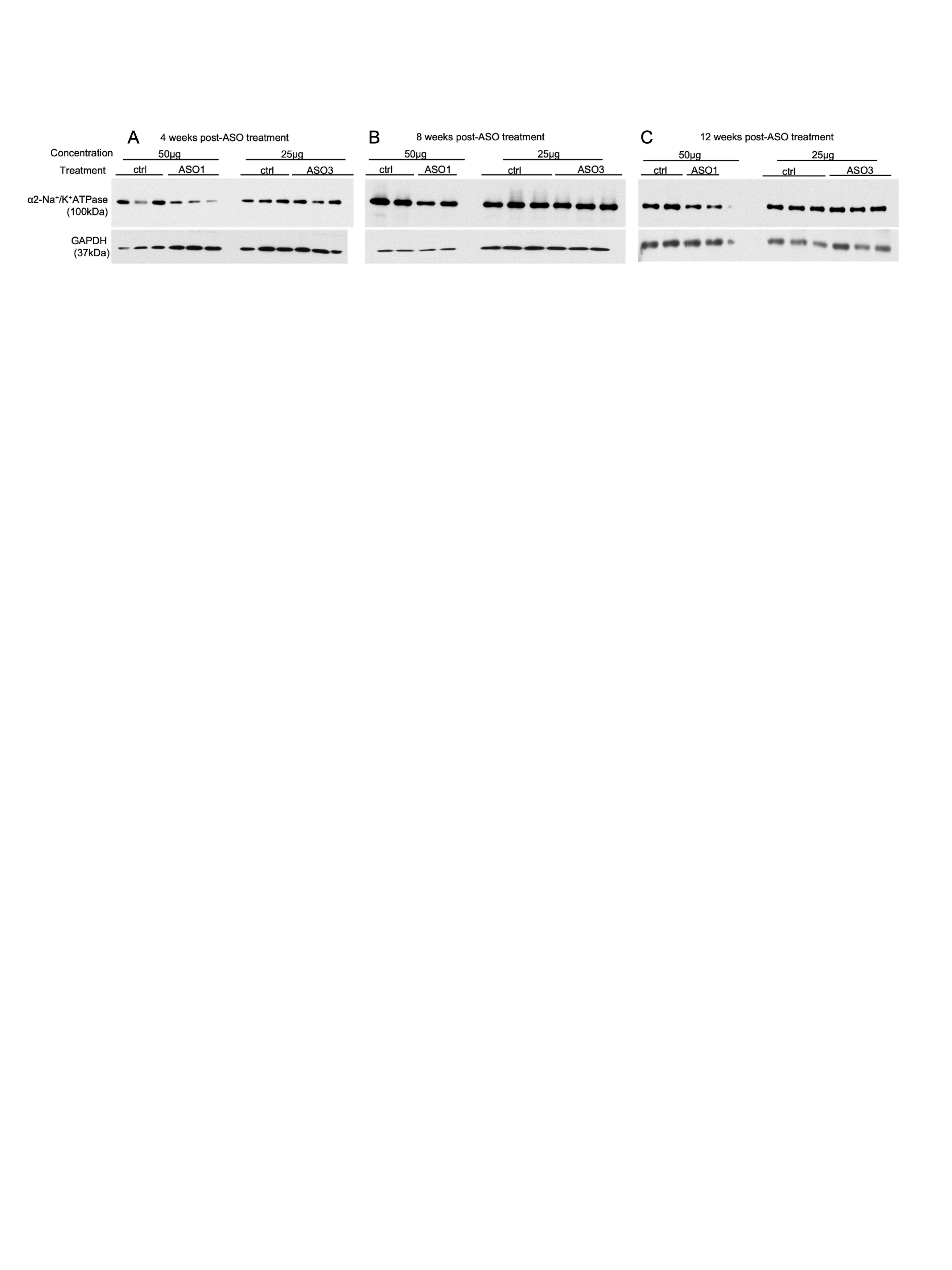

Supplement: S3 Fig — Immunoblot images of α2-Na+/K+-ATPase protein levels in ctrl, Atp1a2 ASO1 or ASO3-treated mouse spinal cord (top lane) and housekeeping protein GAPDH (bottom lane) at (A) 4 weeks, (B) 8 weeks, or (C) 12 weeks post-ASO treatment. (TIF) [file pone.0294731.s003.tif]

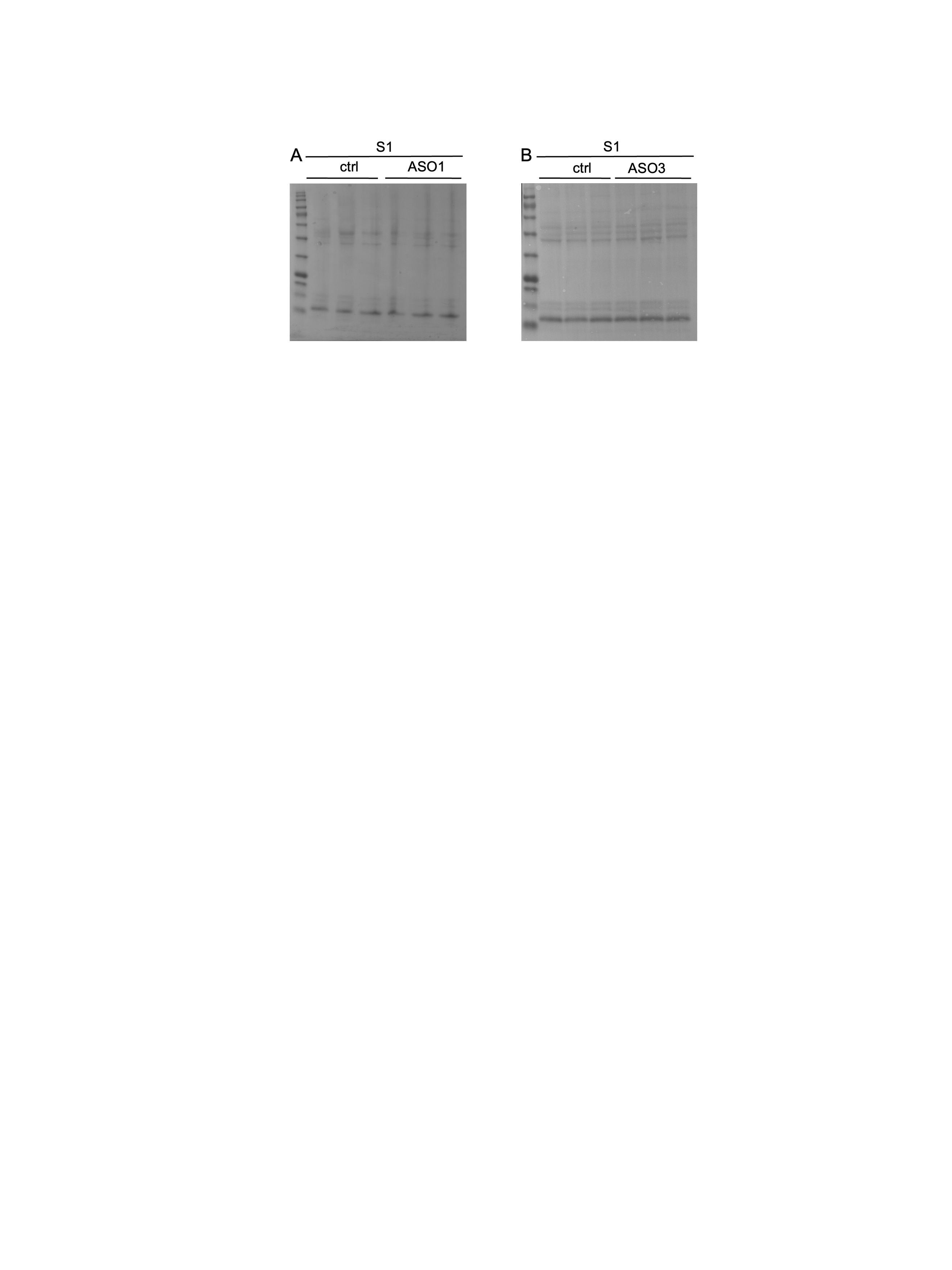

Supplement: S4 Fig — (TIF) [file pone.0294731.s004.tif]

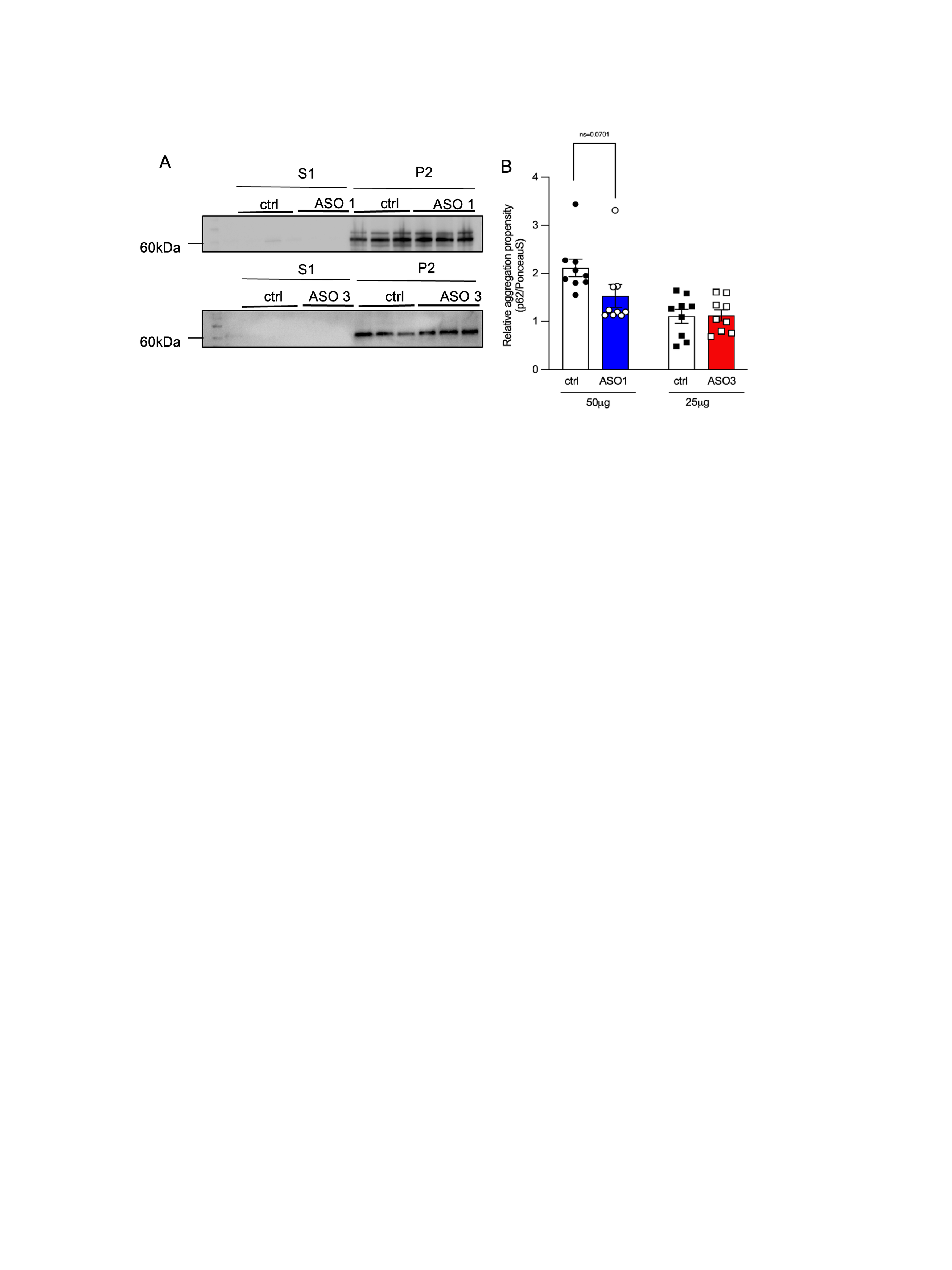

Supplement: S5 Fig — (A) Representative immunoblots and (B) quantitation of the amount of p62 in detergent soluble (S1) and insoluble fractions (P2) of spinal cords from end-stage SOD1*G93A mice treated with single dose ICV injection of ctrl ASO, Atp1a2 ASO1 (A, top panel) or ASO3 (A, bottom panel). N = 9 mice per group. ns = not significant by unpaired t-test with Welch’s correction. (TIF) [file pone.0294731.s005.tif]

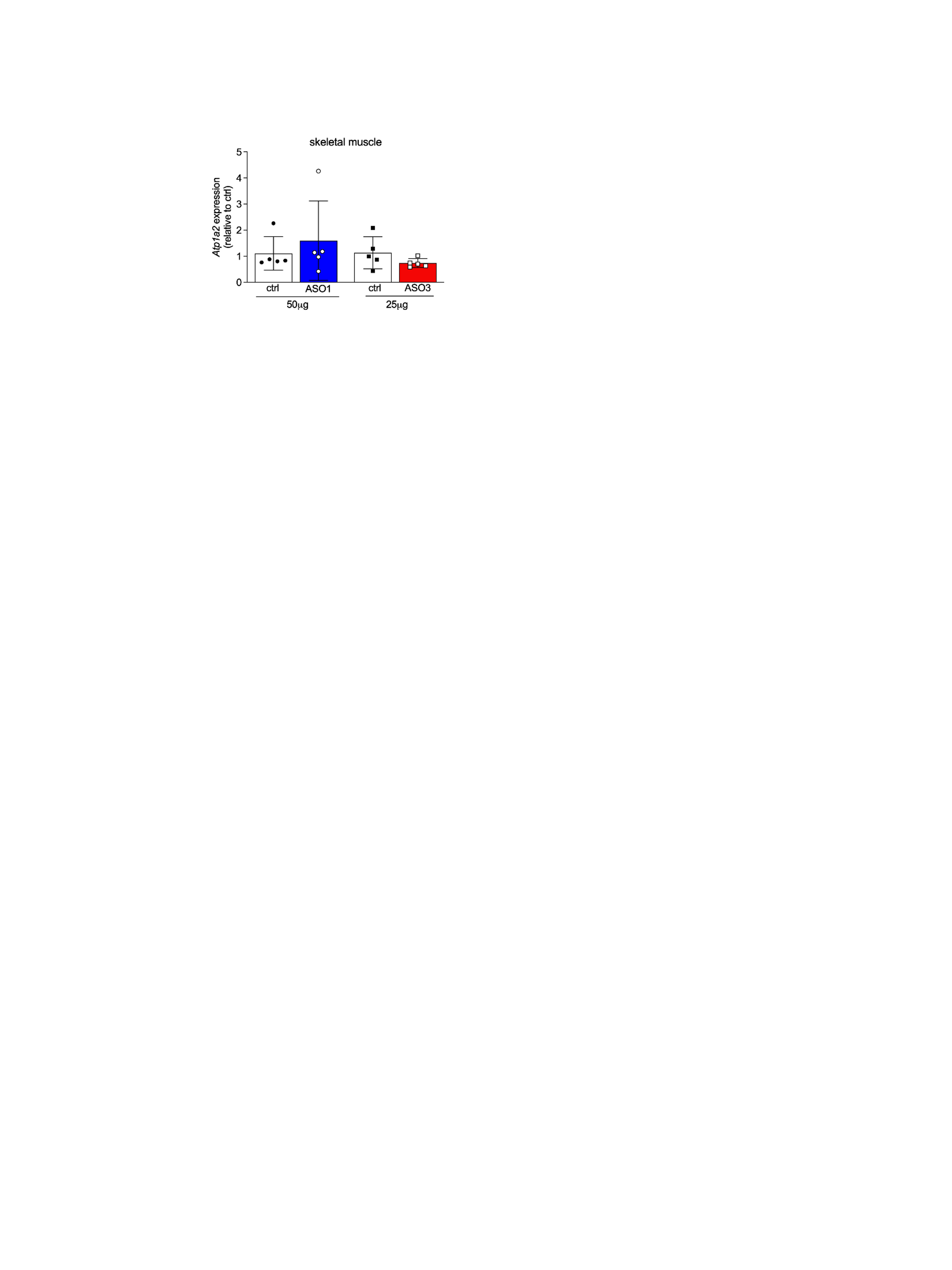

Supplement: S6 Fig — Relative Atp1a2 transcript levels in skeletal muscle (n = 5 mice/group from a subset of SOD1*G93A mice shown in Fig 4, molecular expression not significant by unpaired t-test with Welch’s correction. (TIF) [file pone.0294731.s006.tif]

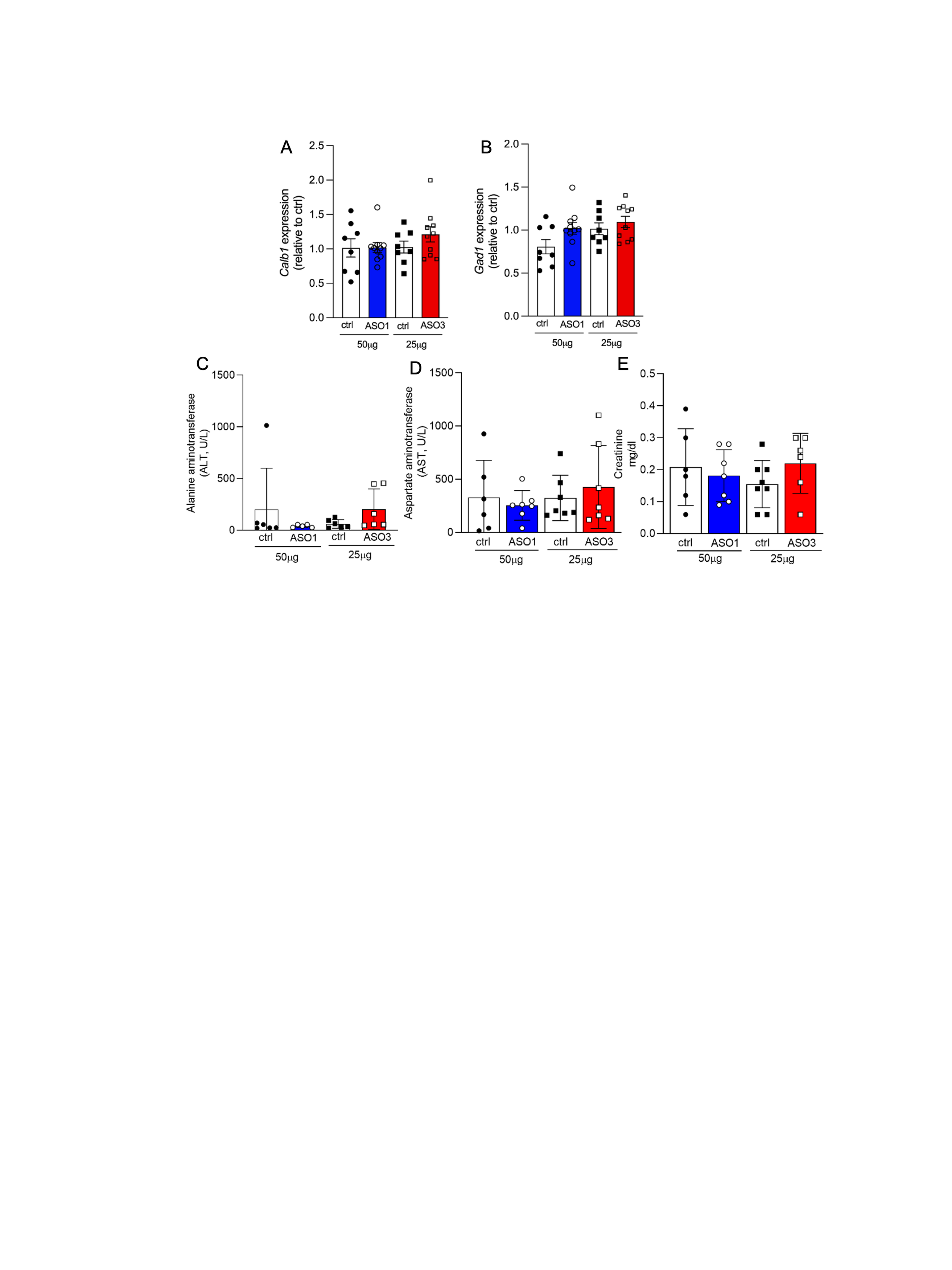

Supplement: S7 Fig — Purkinje cell markers, (A) Calb1 and (B) Gad1 in cerebellum lysates from a subset of SOD1*G93A mice shown in Figs 4 and 5 (n = 8–10 mice/group) were measured by qRT-PCR. Serum measurements of (C) alanine aminotransferase (ALT), (D) aspartate aminotransferase (AST) enzymes and creatinine (E), for signs of liver and kidney toxicity respectively, in a subset of end-stage SOD1*G93A mice shown in Fig 4 (n = 5–7 mice/group). Molecular expression (A, B) and serum levels of markers (C, D, E) not significant by unpaired t-test with Welch’s correction. (TIF) [file pone.0294731.s007.tif]

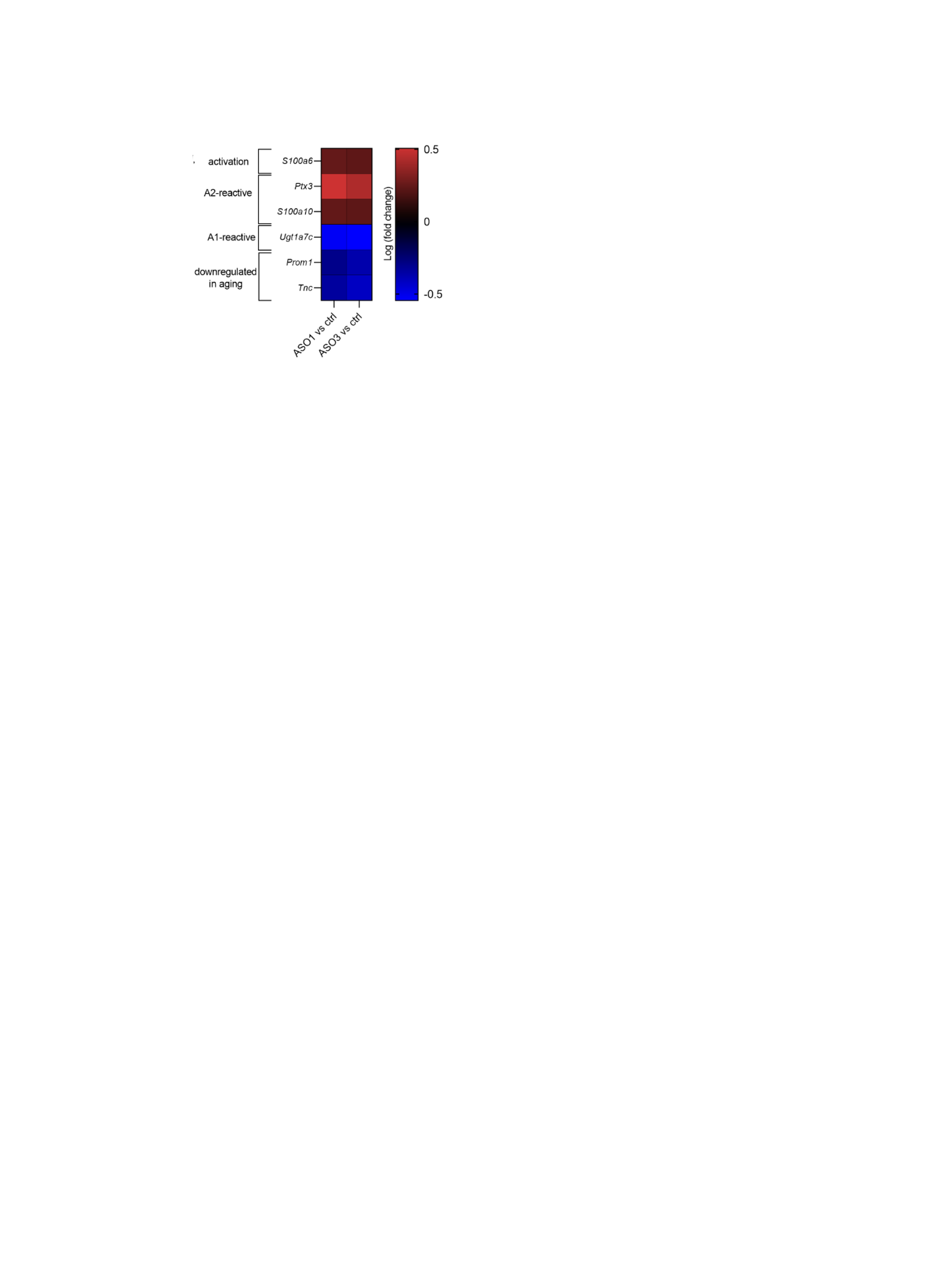

Supplement: S8 Fig — Heat map of genes impacted by ASO treatment and intersecting with astrocyte activation, reactivity, and aging. (TIF) [file pone.0294731.s008.tif]

Fig.2C

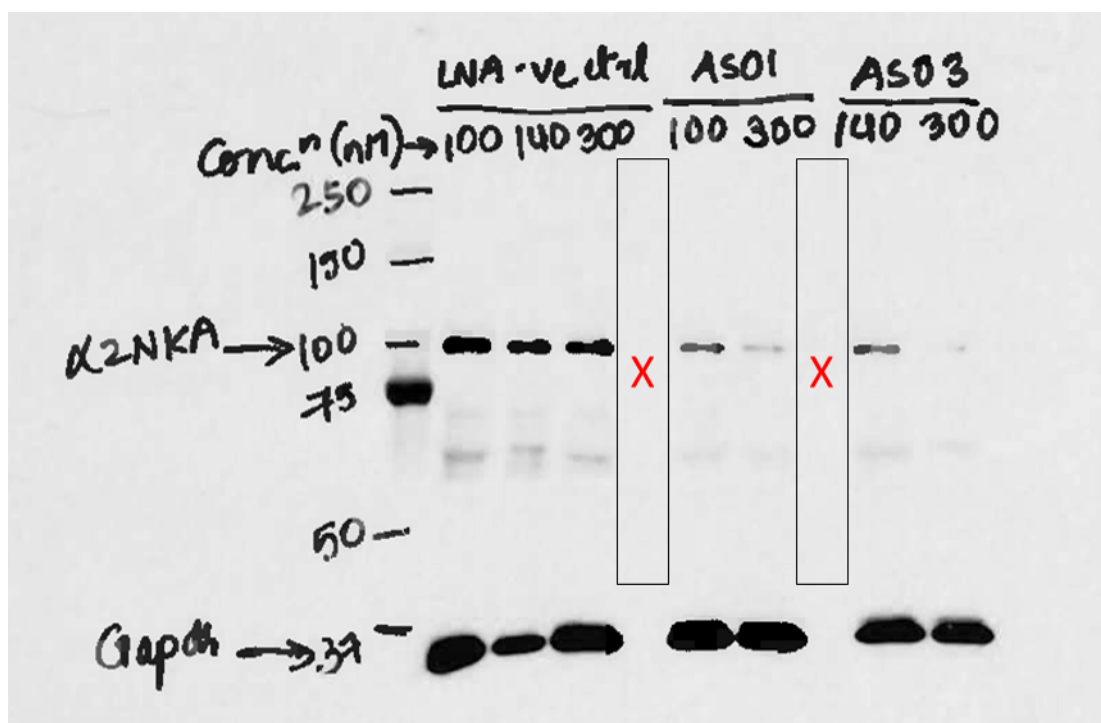

Fig4C\_ASO1

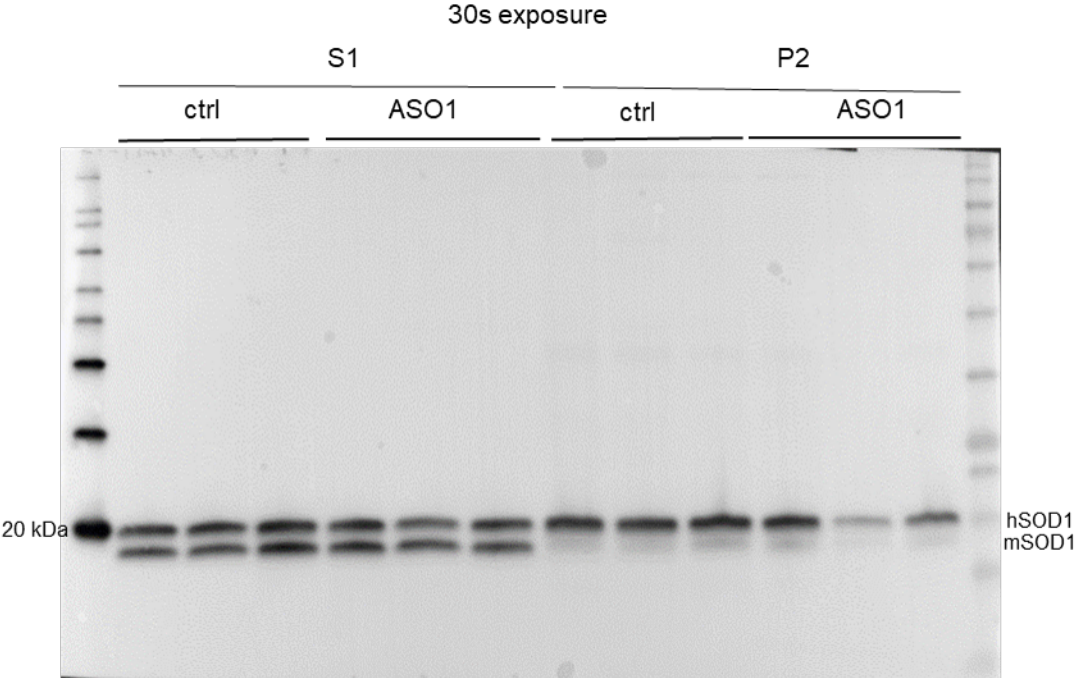

Fig4C\_ASO3

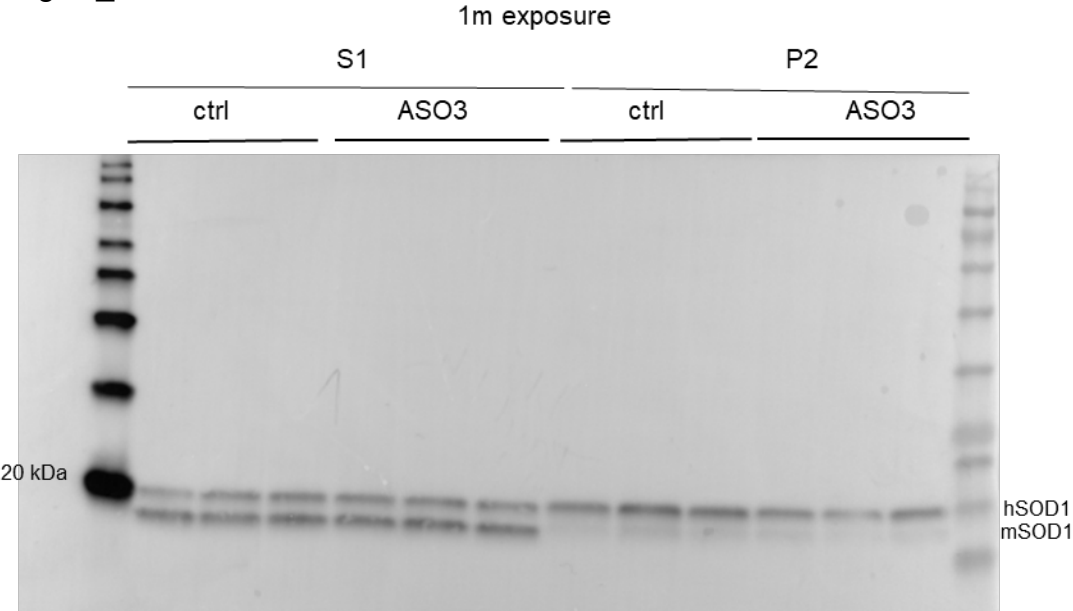

S3A\_Fig

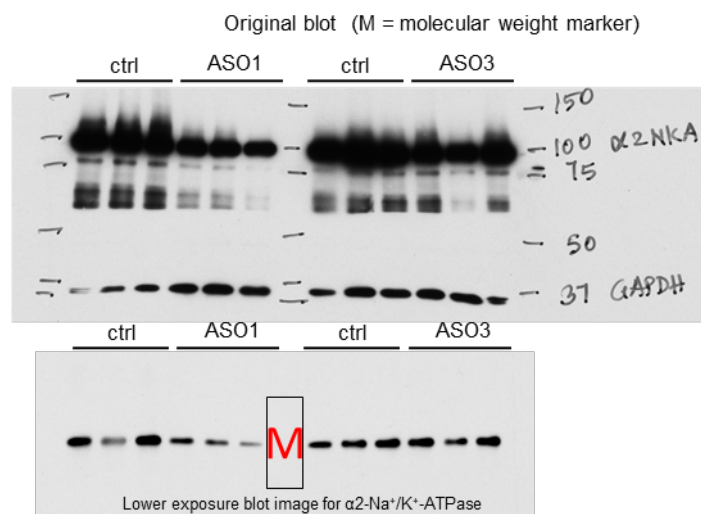

S3B\_Fig

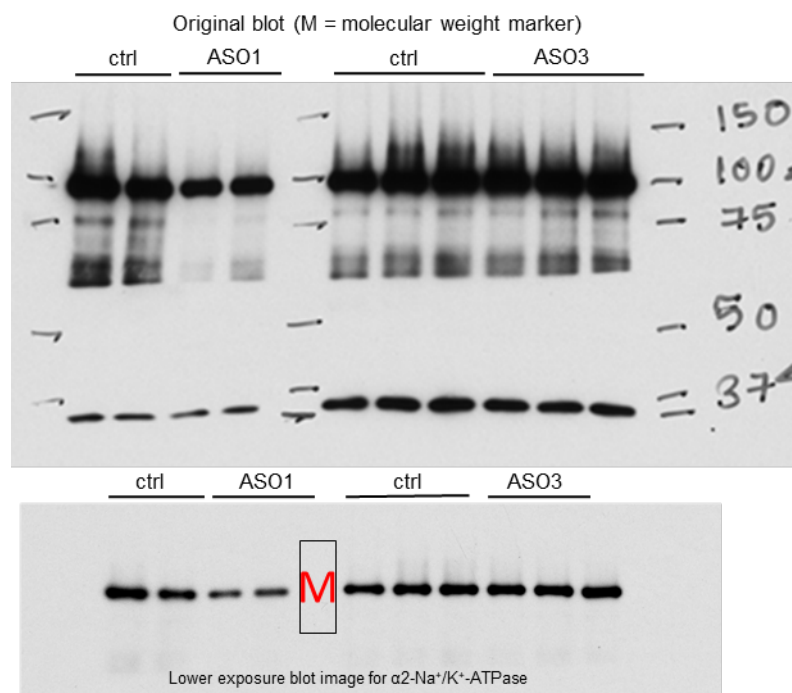

S3C\_Fig

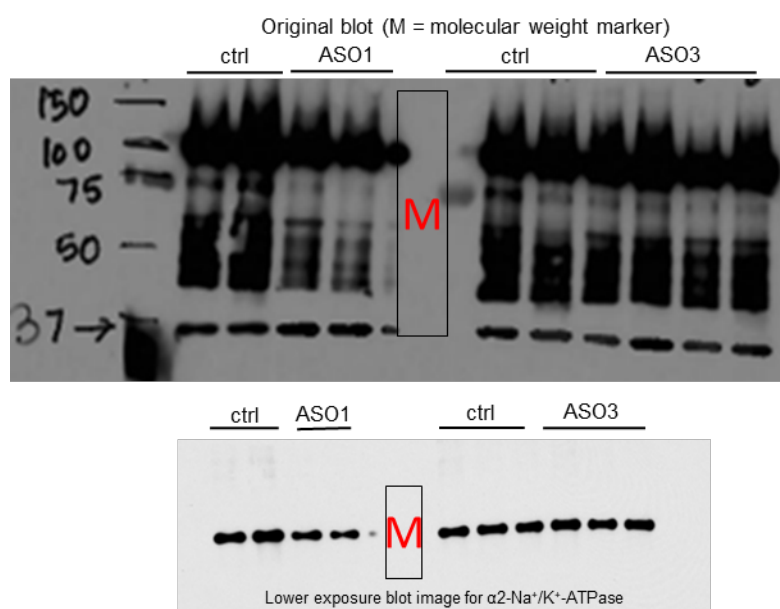

S5 ASO1\_Fig

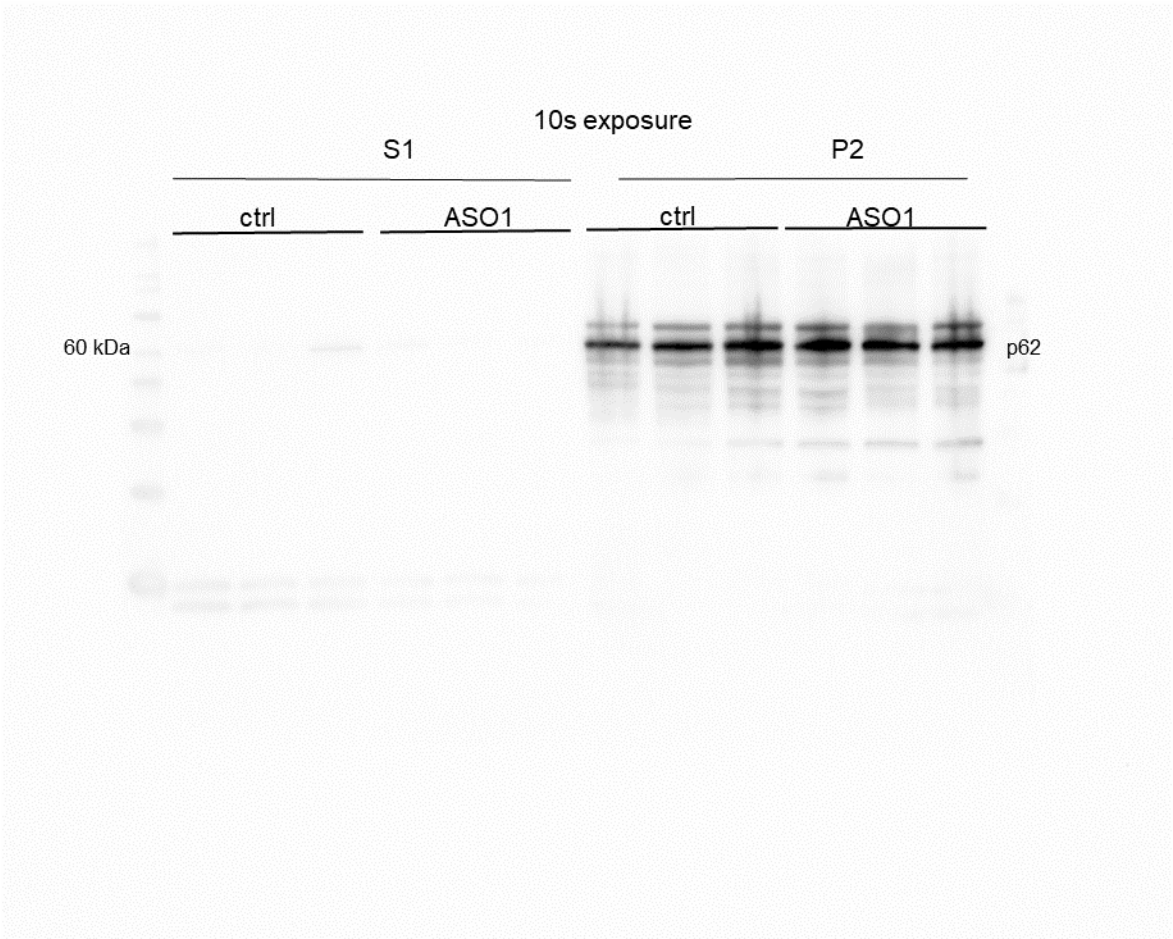

S5\_ASO3\_Fig

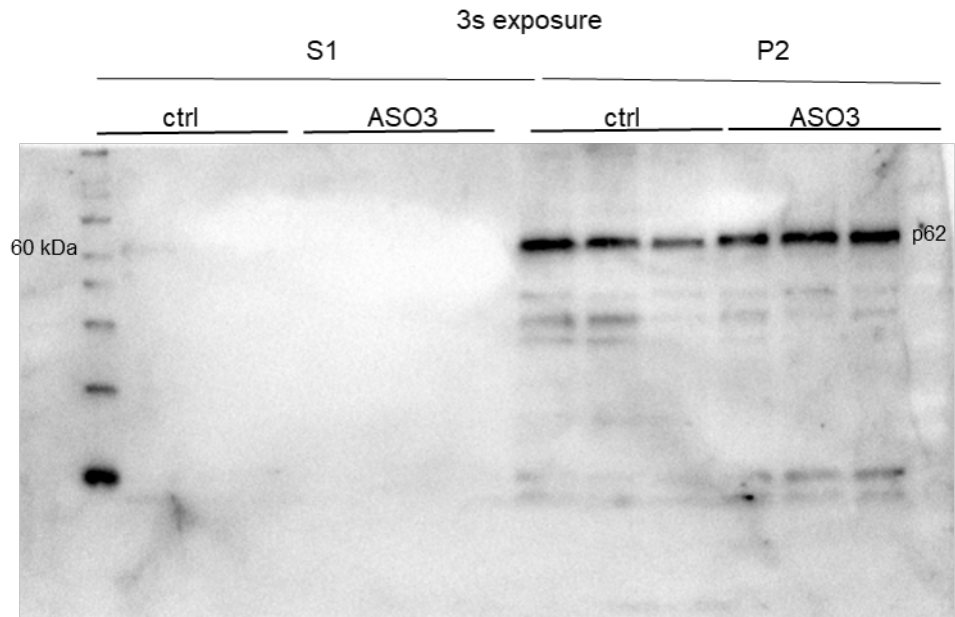

Supplement: S1 Raw images — (PDF) [file pone.0294731.s009.pdf]
